# Language Dominance in Left-Handers: Unveiling Left Hemisphere Global Dominance With Specific Right Hemisphere Regional Dominance

**DOI:** 10.7759/cureus.74691

**Published:** 2024-11-28

**Authors:** Mudathir Bakhit, Ryo Hiruta, Yousuke Kuromi, Satoshi Maesawa, Masazumi Fujii

**Affiliations:** 1 Department of Neurosurgery, Fukushima Medical University, Fukushima, JPN; 2 Department of Neurosurgery/Department of Operation, National Health Organization, Nagoya Medical Center, Nagoya, JPN

**Keywords:** cognitive functions, cortical parcellation, fmri, handedness, hemispheric asymmetry, language

## Abstract

Introduction

The degree to which each human brain hemisphere governs specific cognitive processes, such as language and handedness (the preference or dominance of one hand over the other), varies across individuals. Research has explored the nature of language laterality in left-handed (LH) individuals, indicating that left-hemisphere dominance for language is commonly observed across both left- and right-handed populations. Advanced imaging techniques, including functional transcranial Doppler sonography and fMRI, have revealed subtle differences in language lateralization between LH and right-handed (RH) individuals, particularly in semantic processing tasks. These findings underscore the complex relationship between handedness and language lateralization. This study investigates the spatial patterns of language lateralization in LH and RH individuals using high-resolution fMRI data and the Human Connectome Project (HCP) multimodal parcellation (MMP).

Method

We utilized pre-processed MRI scans from the HCP database, comprising 140 healthy young adults, with 70 individuals in each of the RH and LH groups. The language task includes two contrasts: the STORY contrast, where participants listened to brief auditory stories compared to a baseline, and the STORY-MATH contrast, where participants listened to stories versus solving addition and subtraction problems. Data processing involved the HCP Pipelines and the MMP atlas was applied for analysis. The Edinburgh Handedness Inventory categorized participants as either LH or RH. For analysis, we focused on the number of brain surface elements (3D surface vertices) with positive elements (PEs) within each brain region, indicating blood-oxygen-level-dependent (BOLD) activity. The study's methodology aimed to quantify and compare PEs across the hemispheres (paired sample) and handedness groups (independent sample), providing insights into language lateralization. Statistical analysis involved Mann-Whitney U tests for differences across gender and handedness groups and robust t-tests for hemispheric dominance. Results were visualized by projecting mean and effect size values onto a 3D brain surface.

Results

The analysis of hemispheric mean differences in PEs revealed robust left hemisphere dominance in both the STORY and STORY-MATH contrasts among the RH group, while the LH group exhibited more balanced activity. Significant variations in PEs were observed across numerous MMP regions, with LH individuals showing pronounced asymmetry in 67 and 76 MMP regions (out of 180 regions) in the STORY and STORY-MATH contrasts, respectively, compared to 83 and 99 regions in RH individuals. Additionally, when comparing LH and RH groups, significant differences in PEs were identified within 14 MMP regions (out of 360 regions), all demonstrating significant asymmetry in LH individuals and primarily located in the right hemisphere (12 regions), notably in the inferior parietal lobule (Brodmann 39 and 40). No differences were found in the STORY-MATH contrast.

Conclusion

We identified hemispheric left-lateralization dominance in brain areas associated with language processing, irrespective of handedness. However, employing multimodal brain parcellation with fMRI language tasks unveiled notable differences in specific regions. Particularly striking was the heightened activity observed in certain right hemisphere regions among LH individuals.

## Introduction

The extent to which each human brain hemisphere dominates different cognitive processes, such as language and handedness (the preference or dominance of one hand over the other), varies. It was Marc Dax who first identified the localization of speech in the left hemisphere of the brain in 1811 through studying aphasia patients. His discovery was further supported by Broca in 1865 [1,2]. Moreover, the interplay between handedness and the lateralization of language cognitive function dates back to the 19th century, as initially documented by Ogle in 1871 and Jackson in 1880. Their reports of left-handed (LH) aphasic patients, attributed to lesions in the right hemisphere, laid the groundwork for understanding the connection between handedness and language lateralization [2,3].

Previously posited, the hypothesis of atypical language laterality towards the right cerebral hemisphere in LH subjects has been a topic of debate within neurological research. A comprehensive meta-analysis conducted by Carey et al. aimed to study this concept, revealing interesting findings contrary to initial conjectures [4]. The meta-analysis consistently showed left-laterality prevalence in both LH and right-handed (RH) individuals across the examined factors. Notably, RH individuals demonstrated higher left-laterality values [4]. In the context of the Wada test factor, the study delineated an 87% left dominance prevalence in RH individuals compared to a 65% left dominance prevalence in LH individuals. Additionally, a collective analysis of various independent studies revealed a consistent trend of LH individuals exhibiting comparatively weaker left-laterality in speech when contrasted with their RH counterparts [5-9]. Contrary to the initial hypothesis of atypical laterality, these investigations suggest a pattern characterized by a diminished left dominance in LH rather than an aberrant right-hemisphere bias.

Further exploration into language lateralization vis-à-vis handedness reveals intricate patterns discernible across specific language components. An investigation delved into the influence of handedness on language lateralization employing functional transcranial Doppler sonography (TDS). The study uncovered left-lateralization in word generation, albeit to a lesser extent during semantic processing, suggesting a nuanced relationship between handedness and specific linguistic tasks [10]. Notably, when contrasted with their RH counterparts, LH individuals exhibited a higher propensity toward atypical or inconsistent lateralization, particularly in semantic processing.

In a separate study utilizing a Chinese semantic task-based fMRI paradigm, Goa et al. observed a divergence in left-lateralization concerning semantics between LH and RH individuals. RH individuals showed a distinct left-lateralized activity pattern in the cerebral cortex, while LH individuals displayed a notably weaker left-lateralization during semantic tasks, further accentuating the disparity between handedness groups [11]. These investigations collectively suggest that LH individuals exhibit a reduced degree of left-lateralization, particularly noticeable during semantic processing, compared to their RH counterparts, underscoring the intricate relationship between handedness and language lateralization across varying linguistic elements [4-11].

LH individuals typically exhibit weaker or atypical left laterality compared to RH, yet clear evidence of atypical asymmetry remains elusive. Prior studies investigating functional laterality, particularly using fMRI analyses, primarily examined larger spatial scales, encompassing entire brain hemispheres. Our study, however, aimed to scrutinize language laterality within handedness groups at a more refined spatial level. Leveraging the innovative Human Connectome Project (HCP) multimodal parcellation (MMP), consisting of 180 brain regions in each hemisphere [12], we ventured into a more granular exploration. This approach allowed for a detailed investigation of cortical language organization, offering insight into subtler nuances of language lateralization in LH and RH.

We used the HCP language fMRI task images featuring a 2-mm isotropic resolution. These images had meticulous inter-individual registration analyses on the cortical surface for enhanced precision. To capture accurate gray matter activations, the researchers computed gray ordinate activations on the cortex surface for each subject, aligning with fMRI's recording of activation in the gray matter [12-14]. The current study utilized two contrasts: STORY (auditory story listening vs. baseline) and STORY-MATH (contrasting auditory story listening with the auditory math task). The inclusion of a math task aimed to provide an attentionally demanding comparison [15,16]. The rationale behind this approach lies in the observation that semantic and episodic memory processes typically occur during passive states. In contrast, active, attentionally demanding tasks, such as math problems, interfere with these passive processes, known as the default mode network [15,17]. Therefore, the STORY-MATH contrast enables a comparison between story comprehension-engaging systems for complex semantic and syntactic integration-and a continuous, attentionally demanding, non-semantic, or shallow-semantic task.

A copy of this article was presented at the 25th Congress of Japan Human Mapping Society (February 24-25, 2023) and at the 81st Annual Meeting of the Japan Neurosurgical Society (September 28-30, 2022).

## Materials and methods

HCP data

Images

The study employs a cross-sectional design using pre-processed MRI scans from the open-access HCP data, including language task fMRI scans of 140 healthy young adults (70 in each of the LH and RH groups) [13,14]. The HCP is a large-scale, open-access research initiative aimed at mapping the neural connections and organization of the human brain. It provides high-resolution neuroimaging data, including structural and functional MRI, diffusion imaging, and behavioral assessments, from a large sample of healthy participants. 

We included only subjects who completed the language fMRI task and had a handedness assessment. We randomly selected 70 RH subjects, as only 70 LH subjects with complete language fMRI data were available. Individuals who did not complete the language task or did not have a handedness assessment were excluded. All individuals who participated in the HCP were educated. In the current study's sample, the mean years of education were 14.5. Due to the nature of the study, the need for ethical approval was waived since the data is open-source.

The fMRI data acquisition parameters were as follows: whole-brain EPI acquisitions were acquired with a 32-channel head coil on a modified 3T Siemens Skyra (Siemens Healthineers, Erlangen, Germany) with TR=720 ms, TE=33.1 ms, flip angle=52 deg, BW=2290 Hz/Px, in-plane FOV=208 × 180 mm, 72 slices, and 2.0 mm isotropic voxels, with a multi-band acceleration factor of 8 [16].

Task fMRI

The language task utilized in the HCP was created by Binder et al. [15,16] and involved two runs, each alternating between four blocks of a story and four blocks of a math task. The task was performed twice, one with right-to-left phase encoding and one with left-to-right phase encoding. The blocks vary in length but were designed so that the math task blocks match the length of the story task blocks, with additional math trials added at the end of the task to complete the 3.8-minute run as needed. During the story blocks, participants listened to brief auditory stories adapted from Aesop's fables, followed by a two-alternative forced-choice question related to the story's topic. The language task comprises two contrasts: the story versus baseline (STORY) and the story versus math (STORY-MATH). The math blocks present trials auditorily and require participants to solve addition and subtraction problems. Each trial displays a series of arithmetic operations, followed by "equals" and two answer choices.

fMRI Data Processing

The data was processed via the HCP Pipelines. For the details of the pipelines, please refer to the study by Glasser et al. [18]. The processed data is in the classical volumetric space (NIFTI format) and the surface (CIFTI grayordinate-based) space. This study used the grayordinate-based dataset, which maps the voxels within the cortical gray matter ribbon onto the native cortical surface. Among the statistical maps available, we used the z-stat maps.

MMP of the Human Brain

**Figure 1:**
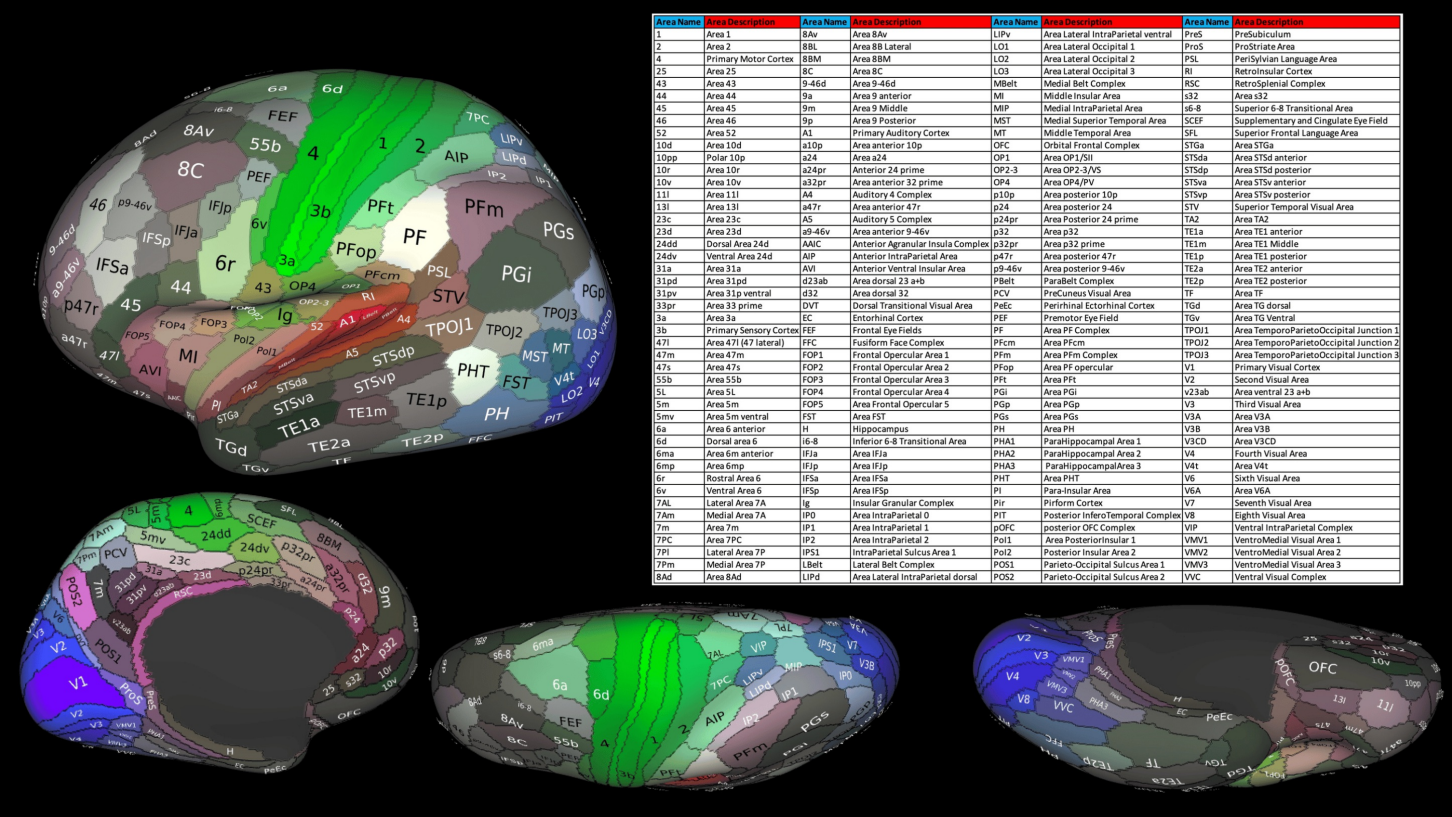
The Human Connectome Project (HCP) multimodal parcellation (MMP). The figure displays the left hemisphere, accompanied by a table describing the labels. Each hemisphere consists of 180 regions. The MMP is available from the HCP database [12]. The data is open-source and available in the Brain Analysis Library of Spatial Maps and Atlases (https://balsa.wustl.edu). To access these open-source data, creating an account is required. The image was created using the Connectome Workbench software, provided as open-source by the HCP (https://www.humanconnectome.org/software/get-connectome-workbench).

The MMP atlas has 180 regions in each hemisphere (Figure 1) [12]. We applied this parcellation to map the language task fMRI activity onto the brain surface, enabling us to compare differences between handedness groups in each MMP region. We also used the same parcellation to visualize our results. The MMP atlas can be accessed through the HCP database website (https://db.humanconnectome.org).

Handedness

The Edinburgh Handedness Inventory (EHI) value of each subject was obtained from the HCP database [19] and categorized as either RHs (positive values) or LHs (negative values). The EHI is a standardized questionnaire used to assess hand preference for tasks like writing, drawing, and throwing. It generates a laterality score ranging from -100 to +100, with -100 indicating strong left-handedness, +100 indicating strong right-handedness, and 0 representing ambidexterity. This tool is widely used in research to classify individuals as left-handed, right-handed, or ambidextrous.

Only 70 LHs with complete language fMRI tasks were found in the HCP database. The EHI values ranged from -40 to -100. However, there was a sufficient sample of RHs with complete fMRI tasks, and all the selected individuals had an EHI of +100. Detailed demographic data, including exact age and handedness, are available only to investigators who agree to the HCP’s Restricted Data Use Terms. Applications can be submitted at https://www.humanconnectome.org/study/hcp-young-adult/document/restricted-data-usage.

Number of positive elements (PEs)

**Figure 2:**
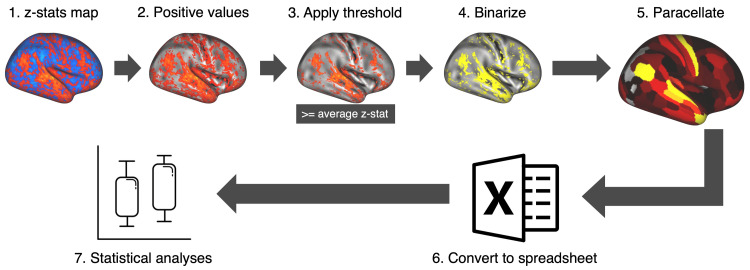
Extracting the number of positive elements (PEs). The process involves the following steps: (1) Downloading z-stat maps from the HCP database. (2) Discarding negative values. (3) Applying a threshold equal to or above the mean value of the z-stat. (4) Converting active vertices to binaries. (5) Converting the number of binaries in each MMP region to a single parcel value. (6) Converting the parcellation files to text spreadsheets. (7) Exporting the spreadsheet to statistical analysis software.

The aim is to quantify PEs, vertices exhibiting a positive blood-oxygen-level-dependent (BOLD) value, within each area of the MMP atlas. This parallels the identification of active voxels in the volumetric fMRI data [20]. Notably, solely PEs with a z-stat value equal to or above the mean across the entire brain surface are included. We executed this methodology by Employing a custom script utilizing HCP workbench commands (wb_command). Figure 2 outlines the procedure employed to tally PE numbers within each MMP region. Describing the intricate functions of these commands extends beyond this study's scope; however, a script template detailing the steps is accessible at https://github.com/MSIB76/PE_numbers. Subsequently, the PE numbers were extracted for further analysis.

Statistical analysis

The descriptive data were presented as the median and interquartile range (IQR). A Mann-Whitney U test was conducted to examine the differences between gender and handedness groups regarding age distribution, average accuracy, and median correct reaction time across conditions in the language task. Confirmation of language hemispheric dominance relied on the robust t-test statistical analyses, incorporating a 20% trimmed mean due to the deviation from normality assumptions [21,22]. The explanatory effect size for the robust t-test (ξ) was generated, where absolute values of ξ = 0.10, 0.30, and 0.50 denoted small, medium, and large effect sizes [21]. To control false discoveries, p-values in all tests were adjusted for multiple comparisons utilizing the Benjamini and Hochberg method, maintaining a false discovery rate (FDR) of 5%. All analyses were executed using the R statistical package 4.0.4 (R Foundation for Statistical Computing, Vienna, Austria; https://www.r-project.org). Violin plots were generated via the ggstatplot R package [23]. The robust statistical analysis was executed via the WRS2 package [21].

Result visualization

The mean and effect size values were projected onto a 3D brain surface utilizing a code (refer to number of positive elements (PEs) subheading) that incorporated the MMP. This process enabled the visualization of these values in relation to the brain's specific regions and boundaries. The resulting representation offered a comprehensive display of the mean and effect size data within the context of the MMP, facilitating a more nuanced understanding of their spatial distribution across the brain.

## Results

Table 1 presents the gender distribution across handedness groups, while Table 2 provides descriptive statistics for age, language task accuracy, and median reaction time within those groups.

**Table 1:** The gender distribution in the handedness groups.

	Group	Female	Male
Gender frequency per handedness group	Left	33	37
	Right	55	15

**Table 2:** The descriptives of the handedness groups. Detailed data, including the exact age, handedness, ethnicity, and race of each participant, are available only to investigators who agree to the Human Connectome Project's (HCP) Restricted Data Use Terms (https://www.humanconnectome.org/study/hcp-young-adult/document/restricted-data-usage). Accordingly, the authors can share sample codes and details with those who provide evidence of permission to access restricted data from the HCP.

	Group	Median	IQR
Age (years)	LEFT	28	5.75
	RIGHT	29.5	6
Average of accuracy from each condition in the language task (%)	LEFT	89.4	9.32
	RIGHT	86.7	8.73
Average of median correct reaction time from each condition in language task (ms)	LEFT	3529.1	309.16
	RIGHT	3552.1	386.78
Accuracy percentage during STORY condition (%)	LEFT	100	12.5
	RIGHT	100	12.5
Median reaction time for correct trials during STORY condition (ms)	LEFT	3264.5	366.63
	RIGHT	3284.4	381.25
Accuracy percentage during Math condition (%)	LEFT	84.2	13.74
	RIGHT	81.9	16.22
Median reaction time for correct trials during Math condition (ms)	LEFT	3771.5	362.69
	RIGHT	3784.3	448.31

There were no statistically significant differences between gender or handedness groups in age distribution, average accuracy, or median correct reaction time across conditions in the language task (p > 0.05; Table 3).

**Table 3:** Differences between gender and handedness groups in age, task accuracy, and median reaction time. All results were analyzed using the Mann-Whitney U test.

Gender Difference	Statistic	p (corrected)	Mean Difference	95% Confidence Interval	
				Lower	Upper
Average of accuracy from each condition in the language task (%)	1987	0.44	-1.62	-4.09	0.893
Average of median correct reaction time from each condition in language task (ms)	2155	0.68	-27.5	-117.75	59.75
Accuracy percentage during STORY condition (%)	2076	0.44	4.48E-05	-1.75e−5	9.28E-06
Median reaction time for correct trials during STORY condition (ms)	2013	0.44	-62.75	-168	40.75
Accuracy percentage during Math condition (%)	1706	0.07	-5	-8.19	-0.694
Median reaction time for correct trials during Math condition (ms)	2281	0.98	-1.7	-101.25	102.5
Handedness Difference					
						Age (years)	2311	0.65	-2.81e−6	-2	1
Average of accuracy from each condition in the language task (%)	2030	0.38	2.3	-0.176	4.762
Average of median correct reaction time from each condition in language task (ms)	2238	0.53	-36.03	-122.625	49.75
Accuracy percentage during STORY condition (%)	2221	0.53	4.44E-05	-8.32e−6	1.80E-05
Median reaction time for correct trials during STORY condition (ms)	2209	0.53	-51.25	-149.5	45.75
Accuracy percentage during Math condition (%)	2065	0.38	2.78	-0.556	6.25
Median reaction time for correct trials during Math condition (ms)	2377	0.76	-16.93	-113.75	76

**Figure 3:**
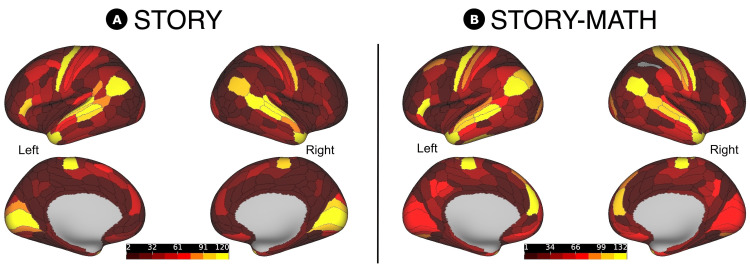
The 20% trimmed mean value of the positive elements (PEs) in each MMP region on both hemispheres. The 20% trimmed mean values of the number of the PEs for the STORY contrast (A) and the STORY-MATH contrast (B) are shown. The upper rows display the lateral surface, while the lower rows show the medial surface of both hemispheres. Color bars represent the 20% trimmed mean of the PEs.

The PEs’ trimmed mean, SE, and 95% CI in each MMP region from the STORY and STORY-MATH contrasts are shown in Table 4. Also, the trimmed mean values are displayed over an inflated brain surface template in Figure 3. The mean value of the PEs of the entire brain surface was 9416.83 ± 256.06 and 12761.65± 298.37 for the STORY and STORY-MATH contrasts. The mean value of the PEs of the entire right hemisphere was 4399.04 ± 146.74 and 6233.62 ± 159.27 for the STORY and STORY-MATH contrasts. The mean value of the PEs of the entire left hemisphere was 5003.11 ± 142.00 and 6588.54 ± 137.13 for the STORY and STORY-MATH contrasts.

**Table 4:** The 20% trimmed mean, SE, and 95% CI of the positive elements' number in each region.

MMP	STORY				STORY-MATH			
	Mean	SE	95% CI		Mean	SE	95% CI	
			Lower	Upper			Lower	Upper
Entire brain surface	9416.83	256.06	8910.52	9923.15	12761.65	298.37	12171.68	13351.63
Right hemisphere	4399.04	146.74	4108.89	4689.18	6233.62	159.27	5918.70	6548.54
Left hemisphere	5003.11	142.00	4722.34	5283.87	6588.54	137.13	6317.39	6859.68
R_V1_ROI	123.93	8.42	107.28	140.58	63.99	5.60	52.92	75.06
L_V1_ROI	116.11	8.38	99.54	132.68	60.54	6.08	48.52	72.55
R_MST_ROI	8.49	1.02	6.46	10.51	27.42	2.02	23.43	31.40
L_MST_ROI	4.01	0.71	2.61	5.41	19.56	1.64	16.32	22.80
R_V6_ROI	4.98	0.79	3.42	6.54	11.51	1.72	8.12	14.91
L_V6_ROI	4.23	0.78	2.68	5.78	8.23	1.59	5.09	11.36
R_V2_ROI	92.48	5.77	81.08	103.88	72.93	5.36	62.33	83.53
L_V2_ROI	80.82	4.95	71.03	90.62	62.81	5.05	52.82	72.80
R_V3_ROI	58.00	3.94	50.20	65.80	71.04	4.55	62.03	80.04
L_V3_ROI	40.70	3.20	34.38	47.03	49.11	3.87	41.45	56.76
R_V4_ROI	53.40	4.35	44.80	62.01	83.29	5.82	71.77	94.80
L_V4_ROI	44.50	3.98	36.63	52.37	80.83	6.53	67.91	93.75
R_V8_ROI	6.75	0.78	5.22	8.28	11.77	1.46	8.89	14.65
L_V8_ROI	3.13	0.48	2.18	4.08	7.96	1.04	5.91	10.02
R_4_ROI	100.55	5.91	88.87	112.23	153.50	10.61	132.51	174.49
L_4_ROI	117.26	6.97	103.49	131.03	117.05	8.12	100.99	133.10
R_3b_ROI	44.01	3.85	36.40	51.63	103.42	8.88	85.86	120.97
L_3b_ROI	57.12	5.38	46.49	67.75	80.10	7.34	65.58	94.61
R_FEF_ROI	18.98	2.07	14.88	23.07	7.27	1.16	4.99	9.56
L_FEF_ROI	23.93	2.05	19.87	27.99	4.68	0.84	3.02	6.34
R_PEF_ROI	8.95	1.12	6.74	11.17	1.46	0.35	0.78	2.15
L_PEF_ROI	8.18	1.06	6.07	10.28	0.62	0.18	0.26	0.98
R_55b_ROI	39.55	2.18	35.24	43.85	19.24	1.94	15.39	23.08
L_55b_ROI	75.27	3.04	69.27	81.28	33.13	1.92	29.33	36.93
R_V3A_ROI	9.36	1.50	6.38	12.33	22.08	3.05	16.06	28.11
L_V3A_ROI	6.62	1.36	3.93	9.30	17.62	2.99	11.70	23.53
R_RSC_ROI	7.86	0.81	6.26	9.46	19.19	1.68	15.87	22.51
L_RSC_ROI	5.76	0.78	4.22	7.30	15.00	1.15	12.72	17.28
R_POS2_ROI	1.57	0.29	1.00	2.14	12.02	1.34	9.38	14.67
L_POS2_ROI	1.10	0.22	0.65	1.54	17.50	1.67	14.19	20.81
R_V7_ROI	2.95	0.56	1.84	4.07	8.20	0.85	6.53	9.87
L_V7_ROI	1.68	0.35	0.98	2.38	6.07	0.96	4.17	7.97
R_IPS1_ROI	9.87	1.29	7.33	12.41	14.82	1.70	11.46	18.18
L_IPS1_ROI	12.07	1.14	9.82	14.33	13.68	1.80	10.13	17.23
R_FFC_ROI	21.96	1.69	18.62	25.31	44.79	2.56	39.72	49.85
L_FFC_ROI	17.92	1.54	14.87	20.96	43.12	2.54	38.11	48.13
R_V3B_ROI	4.95	0.89	3.19	6.71	13.24	1.69	9.89	16.59
L_V3B_ROI	4.67	0.89	2.91	6.43	12.48	1.80	8.92	16.03
R_LO1_ROI	5.37	0.76	3.87	6.86	13.83	1.33	11.20	16.46
L_LO1_ROI	2.55	0.39	1.77	3.33	8.98	1.21	6.58	11.37
R_LO2_ROI	3.99	0.55	2.90	5.08	12.39	1.11	10.19	14.59
L_LO2_ROI	3.92	0.72	2.49	5.34	15.73	1.50	12.76	18.70
R_PIT_ROI	7.00	0.85	5.32	8.68	17.10	1.52	14.10	20.09
L_PIT_ROI	3.51	0.61	2.31	4.72	14.02	1.31	11.43	16.62
R_MT_ROI	4.76	0.57	3.64	5.88	12.60	1.10	10.43	14.77
L_MT_ROI	4.63	0.69	3.27	5.99	16.74	1.32	14.12	19.36
R_A1_ROI	47.20	1.31	44.61	49.79	20.56	1.63	17.34	23.78
L_A1_ROI	54.01	1.63	50.78	57.24	21.98	1.67	18.68	25.27
R_PSL_ROI	23.25	2.73	17.86	28.64	14.04	1.71	10.66	17.41
L_PSL_ROI	76.89	4.70	67.60	86.18	35.95	2.72	30.56	41.34
R_SFL_ROI	23.06	1.72	19.66	26.46	28.51	1.90	24.75	32.28
L_SFL_ROI	61.52	3.38	54.84	68.20	48.25	2.46	43.39	53.11
R_PCV_ROI	12.58	1.72	9.17	15.99	22.85	2.24	18.42	27.27
L_PCV_ROI	12.69	1.26	10.20	15.18	26.94	2.09	22.81	31.07
R_STV_ROI	18.81	2.03	14.79	22.83	30.51	2.45	25.67	35.35
L_STV_ROI	33.18	2.32	28.58	37.77	49.26	2.09	45.12	53.40
R_7Pm_ROI	9.64	1.20	7.27	12.02	1.06	0.29	0.49	1.63
L_7Pm_ROI	7.23	0.89	5.46	8.99	1.10	0.29	0.53	1.66
R_7m_ROI	20.99	2.37	16.31	25.67	68.36	2.86	62.71	74.01
L_7m_ROI	21.25	1.95	17.40	25.10	67.10	2.49	62.18	72.01
R_POS1_ROI	14.35	1.73	10.93	17.76	31.15	2.75	25.72	36.59
L_POS1_ROI	11.29	1.26	8.79	13.78	39.87	3.17	33.61	46.13
R_23d_ROI	2.87	0.57	1.75	3.99	8.05	1.00	6.07	10.02
L_23d_ROI	1.62	0.30	1.03	2.20	9.18	0.76	7.68	10.68
R_v23ab_ROI	5.14	0.78	3.60	6.69	38.50	1.80	34.95	42.05
L_v23ab_ROI	2.81	0.51	1.81	3.81	28.79	1.80	25.22	32.35
R_d23ab_ROI	1.25	0.29	0.68	1.82	7.42	0.93	5.57	9.26
L_d23ab_ROI	3.48	0.57	2.35	4.60	25.13	1.26	22.65	27.62
R_31pv_ROI	4.83	0.69	3.47	6.20	46.14	3.02	40.18	52.11
L_31pv_ROI	5.60	0.76	4.09	7.10	57.90	2.46	53.05	62.76
R_5m_ROI	13.88	1.33	11.25	16.51	29.93	2.56	24.87	34.99
L_5m_ROI	7.35	0.92	5.53	9.16	18.18	1.89	14.44	21.92
R_5mv_ROI	14.86	1.66	11.58	18.14	29.82	3.28	23.34	36.30
L_5mv_ROI	9.86	1.36	7.16	12.55	24.13	2.58	19.04	29.22
R_23c_ROI	12.62	1.43	9.80	15.44	22.15	2.50	17.21	27.10
L_23c_ROI	12.74	1.44	9.89	15.59	22.77	2.68	17.47	28.08
R_5L_ROI	15.38	1.38	12.65	18.11	34.62	3.11	28.47	40.77
L_5L_ROI	12.44	1.34	9.79	15.09	31.37	2.59	26.25	36.49
R_24dd_ROI	29.42	2.02	25.42	33.42	45.98	3.29	39.47	52.48
L_24dd_ROI	36.00	2.46	31.14	40.86	32.88	2.18	28.57	37.19
R_24dv_ROI	15.42	1.36	12.72	18.11	30.73	2.30	26.18	35.27
L_24dv_ROI	12.26	1.28	9.73	14.80	30.54	2.54	25.51	35.56
R_7AL_ROI	8.50	1.28	5.97	11.03	35.74	3.77	28.27	43.20
L_7AL_ROI	10.31	1.53	7.29	13.33	33.81	3.58	26.74	40.88
R_SCEF_ROI	31.96	2.12	27.77	36.16	6.75	0.83	5.11	8.39
L_SCEF_ROI	41.25	2.83	35.65	46.85	4.93	0.62	3.71	6.15
R_6ma_ROI	10.93	1.13	8.69	13.17	7.00	0.95	5.12	8.88
L_6ma_ROI	10.51	1.32	7.91	13.12	5.93	0.82	4.31	7.55
R_7Am_ROI	11.65	1.50	8.70	14.61	13.85	2.21	9.48	18.21
L_7Am_ROI	8.36	1.43	5.53	11.18	11.70	1.56	8.62	14.79
R_7PL_ROI	8.18	1.23	5.74	10.62	0.51	0.23	0.05	0.97
L_7PL_ROI	9.70	1.34	7.05	12.36	0.52	0.23	0.07	0.98
R_7PC_ROI	15.02	1.79	11.49	18.56	25.07	2.68	19.78	30.37
L_7PC_ROI	16.64	2.11	12.47	20.82	15.04	1.68	11.71	18.36
R_LIPv_ROI	10.94	1.41	8.15	13.73	15.40	1.93	11.59	19.22
L_LIPv_ROI	11.10	1.36	8.40	13.79	5.62	0.97	3.71	7.53
R_VIP_ROI	14.40	1.54	11.35	17.46	12.42	1.42	9.62	15.22
L_VIP_ROI	14.25	1.69	10.91	17.59	7.65	1.10	5.49	9.82
R_MIP_ROI	18.38	1.77	14.88	21.88	2.61	0.51	1.60	3.62
L_MIP_ROI	14.40	1.62	11.20	17.61	1.35	0.46	0.43	2.26
R_1_ROI	33.48	2.62	28.29	38.66	90.82	5.85	79.26	102.38
L_1_ROI	46.51	4.91	36.80	56.22	50.37	3.94	42.58	58.16
R_2_ROI	65.35	6.28	52.93	77.76	106.86	8.19	90.66	123.05
L_2_ROI	64.60	7.20	50.37	78.82	65.45	6.31	52.98	77.92
R_3a_ROI	35.43	3.38	28.74	42.12	55.87	4.80	46.37	65.37
L_3a_ROI	41.83	3.23	35.44	48.23	38.18	3.65	30.97	45.39
R_6d_ROI	22.32	2.05	18.26	26.38	21.14	2.20	16.79	25.49
L_6d_ROI	23.65	2.56	18.59	28.72	12.06	1.48	9.13	14.99
R_6mp_ROI	20.31	1.97	16.41	24.21	45.36	3.89	37.66	53.05
L_6mp_ROI	21.64	2.19	17.30	25.98	35.74	3.01	29.79	41.69
R_6v_ROI	16.88	1.46	13.99	19.77	10.88	1.23	8.46	13.31
L_6v_ROI	17.33	1.60	14.17	20.50	3.74	0.73	2.30	5.17
R_p24pr_ROI	6.56	0.64	5.30	7.82	11.19	1.14	8.94	13.44
L_p24pr_ROI	7.82	0.76	6.33	9.32	16.12	1.15	13.85	18.39
R_33pr_ROI	6.11	0.51	5.09	7.12	2.00	0.39	1.22	2.78
L_33pr_ROI	4.73	0.54	3.66	5.80	1.80	0.35	1.11	2.49
R_a24pr_ROI	2.42	0.46	1.51	3.32	3.32	0.50	2.33	4.32
L_a24pr_ROI	4.63	0.72	3.21	6.05	7.08	1.03	5.05	9.12
R_p32pr_ROI	11.96	1.56	8.89	15.04	3.54	0.63	2.30	4.77
L_p32pr_ROI	12.57	1.17	10.27	14.88	3.29	0.52	2.26	4.31
R_a24_ROI	4.26	0.49	3.29	5.23	17.61	1.01	15.60	19.61
L_a24_ROI	5.11	0.44	4.23	5.98	21.82	1.16	19.54	24.11
R_d32_ROI	4.55	0.61	3.34	5.76	4.19	0.69	2.83	5.55
L_d32_ROI	7.56	0.87	5.84	9.28	12.18	1.25	9.70	14.66
R_8BM_ROI	25.01	1.80	21.46	28.57	11.11	1.11	8.91	13.30
L_8BM_ROI	30.26	2.59	25.14	35.38	21.42	1.44	18.56	24.27
R_p32_ROI	2.65	0.50	1.67	3.64	14.79	1.11	12.58	16.99
L_p32_ROI	2.79	0.44	1.93	3.65	15.77	1.16	13.47	18.07
R_10r_ROI	5.54	0.54	4.46	6.61	34.49	1.23	32.05	36.93
L_10r_ROI	8.57	0.72	7.15	9.99	41.74	1.85	38.07	45.40
R_47m_ROI	6.58	0.79	5.03	8.14	33.35	1.42	30.55	36.14
L_47m_ROI	6.31	0.56	5.21	7.41	30.51	1.33	27.89	33.13
R_8Av_ROI	15.33	1.76	11.85	18.82	42.92	3.29	36.41	49.42
L_8Av_ROI	33.98	2.55	28.94	39.02	87.24	3.30	80.70	93.77
R_8Ad_ROI	12.37	1.43	9.55	15.19	56.38	3.00	50.45	62.31
L_8Ad_ROI	15.71	1.53	12.68	18.75	61.11	3.35	54.48	67.73
R_9m_ROI	36.58	2.42	31.79	41.37	110.18	3.64	102.98	117.37
L_9m_ROI	54.36	3.35	47.73	60.98	154.17	3.79	146.68	161.66
R_8BL_ROI	24.62	2.26	20.15	29.09	90.76	3.57	83.70	97.83
L_8BL_ROI	26.58	2.07	22.49	30.68	87.19	2.62	82.01	92.37
R_9p_ROI	5.29	0.72	3.87	6.71	25.79	1.91	22.01	29.56
L_9p_ROI	16.05	1.76	12.57	19.52	65.32	2.61	60.15	70.49
R_10d_ROI	8.05	0.79	6.48	9.62	38.30	2.14	34.06	42.53
L_10d_ROI	11.50	1.17	9.19	13.81	52.90	2.36	48.24	57.57
R_8C_ROI	36.21	2.97	30.34	42.09	37.02	3.79	29.53	44.52
L_8C_ROI	48.62	4.32	40.07	57.16	44.32	3.78	36.85	51.79
R_44_ROI	28.43	2.24	24.01	32.85	22.82	1.80	19.26	26.39
L_44_ROI	56.42	3.34	49.80	63.03	37.27	2.13	33.06	41.48
R_45_ROI	38.43	2.23	34.03	42.83	76.31	2.06	72.24	80.38
L_45_ROI	90.36	3.56	83.32	97.40	130.44	2.88	124.75	136.13
R_47l_ROI	27.85	1.71	24.47	31.23	63.11	2.39	58.38	67.83
L_47l_ROI	47.73	2.25	43.28	52.17	81.12	1.67	77.82	84.42
R_a47r_ROI	12.42	1.05	10.34	14.49	22.92	1.67	19.61	26.22
L_a47r_ROI	22.95	2.11	18.78	27.12	48.81	2.68	43.52	54.10
R_6r_ROI	19.83	2.01	15.85	23.82	10.73	1.33	8.10	13.35
L_6r_ROI	28.50	2.70	23.16	33.84	7.50	1.05	5.42	9.58
R_IFJa_ROI	20.83	1.73	17.41	24.25	16.45	1.33	13.83	19.08
L_IFJa_ROI	31.56	2.14	27.32	35.80	16.02	1.92	12.23	19.82
R_IFJp_ROI	6.31	0.94	4.45	8.17	0.95	0.30	0.36	1.54
L_IFJp_ROI	13.36	1.42	10.54	16.17	0.50	0.24	0.03	0.97
R_IFSp_ROI	24.37	1.90	20.60	28.13	41.50	2.05	37.44	45.56
L_IFSp_ROI	36.06	2.26	31.59	40.53	38.05	1.75	34.60	41.50
R_IFSa_ROI	6.67	0.90	4.88	8.45	31.57	2.01	27.60	35.54
L_IFSa_ROI	16.06	1.44	13.21	18.91	26.29	1.73	22.87	29.70
R_p9_46v_ROI	11.65	1.45	8.79	14.52	1.32	0.29	0.75	1.90
L_p9_46v_ROI	13.24	1.59	10.09	16.39	0.73	0.23	0.27	1.18
R_46_ROI	8.15	1.27	5.63	10.68	5.35	0.87	3.63	7.06
L_46_ROI	7.45	1.32	4.84	10.06	10.13	1.26	7.65	12.62
R_a9_46v_ROI	2.74	0.49	1.76	3.72	1.13	0.29	0.56	1.71
L_a9_46v_ROI	5.26	0.79	3.70	6.83	2.00	0.45	1.11	2.89
R_9_46d_ROI	8.67	1.03	6.63	10.71	6.79	0.87	5.07	8.50
L_9_46d_ROI	15.64	1.89	11.91	19.38	7.67	0.90	5.89	9.44
R_9a_ROI	9.35	1.16	7.05	11.64	43.80	2.57	38.72	48.88
L_9a_ROI	20.02	1.74	16.59	23.46	66.18	2.89	60.47	71.89
R_10v_ROI	14.83	1.01	12.83	16.83	57.46	1.96	53.60	61.33
L_10v_ROI	22.40	1.41	19.62	25.19	69.73	2.02	65.73	73.72
R_a10p_ROI	3.77	0.55	2.68	4.87	5.39	0.83	3.75	7.03
L_a10p_ROI	7.93	0.99	5.97	9.89	12.25	1.51	9.26	15.24
R_10pp_ROI	10.77	0.80	9.20	12.35	21.35	1.40	18.59	24.11
L_10pp_ROI	11.21	0.89	9.46	12.97	20.02	1.52	17.01	23.03
R_11l_ROI	12.13	1.08	10.00	14.26	11.37	1.01	9.36	13.37
L_11l_ROI	9.44	0.90	7.66	11.22	15.18	1.22	12.76	17.60
R_13l_ROI	9.26	0.85	7.57	10.95	21.96	1.51	18.99	24.94
L_13l_ROI	10.94	0.88	9.21	12.67	21.14	1.80	17.59	24.69
R_OFC_ROI	52.69	1.98	48.77	56.61	68.37	2.72	62.99	73.75
L_OFC_ROI	44.00	1.78	40.48	47.52	51.37	3.05	45.33	57.40
R_47s_ROI	23.02	1.57	19.92	26.13	39.29	1.78	35.76	42.81
L_47s_ROI	18.18	1.37	15.47	20.89	40.46	1.82	36.87	44.06
R_LIPd_ROI	4.44	0.80	2.86	6.02	0.00	0.00	0.00	0.00
L_LIPd_ROI	6.86	0.99	4.90	8.82	0.00	0.00	0.00	0.00
R_6a_ROI	36.29	3.77	28.84	43.73	11.77	1.38	9.04	14.51
L_6a_ROI	42.14	3.77	34.70	49.59	13.21	1.61	10.03	16.40
R_i6_8_ROI	6.54	0.94	4.67	8.40	1.00	0.24	0.53	1.47
L_i6_8_ROI	7.92	0.89	6.16	9.67	2.85	0.52	1.82	3.87
R_s6_8_ROI	4.04	0.57	2.91	5.16	5.68	0.89	3.93	7.43
L_s6_8_ROI	1.82	0.33	1.16	2.48	1.64	0.34	0.96	2.32
R_43_ROI	16.73	1.56	13.63	19.82	36.57	2.14	32.33	40.81
L_43_ROI	17.02	1.56	13.95	20.10	26.32	2.47	21.44	31.20
R_OP4_ROI	36.87	2.17	32.59	41.15	55.35	3.74	47.96	62.73
L_OP4_ROI	39.46	2.79	33.95	44.98	34.38	3.07	28.31	40.45
R_OP1_ROI	13.67	1.04	11.61	15.73	22.61	1.97	18.71	26.50
L_OP1_ROI	22.98	2.04	18.94	27.01	22.92	2.31	18.36	27.48
R_OP2_3_ROI	8.14	0.99	6.19	10.09	32.77	2.35	28.13	37.42
L_OP2_3_ROI	17.26	1.46	14.37	20.16	37.70	3.00	31.78	43.63
R_52_ROI	9.05	0.91	7.24	10.85	6.17	0.82	4.54	7.80
L_52_ROI	13.50	1.37	10.80	16.20	10.77	1.18	8.43	13.11
R_RI_ROI	58.82	3.81	51.28	66.36	46.94	3.91	39.20	54.68
L_RI_ROI	65.26	3.28	58.77	71.75	31.98	2.83	26.38	37.57
R_PFcm_ROI	16.06	1.75	12.60	19.52	57.65	4.21	49.34	65.97
L_PFcm_ROI	13.74	1.47	10.84	16.64	27.82	2.71	22.47	33.17
R_PoI2_ROI	12.42	1.38	9.69	15.14	28.58	2.21	24.22	32.95
L_PoI2_ROI	11.68	0.98	9.75	13.61	21.76	1.89	18.02	25.50
R_TA2_ROI	43.63	1.90	39.87	47.39	32.10	1.73	28.67	35.52
L_TA2_ROI	34.17	1.32	31.55	36.78	26.08	1.38	23.36	28.81
R_FOP4_ROI	14.17	1.24	11.72	16.61	2.05	0.40	1.26	2.83
L_FOP4_ROI	23.13	2.13	18.92	27.34	4.14	0.61	2.95	5.34
R_MI_ROI	4.26	0.61	3.06	5.46	3.30	0.47	2.38	4.22
L_MI_ROI	6.83	0.81	5.24	8.43	4.57	0.67	3.25	5.90
R_Pir_ROI	30.42	2.04	26.38	34.45	53.52	2.90	47.79	59.26
L_Pir_ROI	28.44	1.79	24.90	31.98	50.27	2.48	45.38	55.17
R_AVI_ROI	20.81	1.79	17.26	24.35	1.60	0.29	1.02	2.17
L_AVI_ROI	18.45	1.60	15.28	21.62	2.15	0.41	1.35	2.96
R_AAIC_ROI	2.60	0.39	1.82	3.37	3.14	0.44	2.27	4.01
L_AAIC_ROI	3.83	0.57	2.72	4.95	4.05	0.59	2.88	5.22
R_FOP1_ROI	5.40	0.56	4.31	6.50	6.92	0.86	5.21	8.62
L_FOP1_ROI	3.18	0.49	2.21	4.15	4.55	0.66	3.24	5.86
R_FOP3_ROI	2.89	0.56	1.78	4.00	14.19	1.49	11.24	17.15
L_FOP3_ROI	3.31	0.55	2.22	4.40	11.79	1.32	9.17	14.40
R_FOP2_ROI	7.86	1.00	5.88	9.83	14.79	1.46	11.90	17.67
L_FOP2_ROI	10.39	1.02	8.37	12.42	8.92	1.15	6.64	11.20
R_PFt_ROI	18.69	2.42	13.90	23.48	25.21	2.82	19.63	30.80
L_PFt_ROI	19.15	2.43	14.35	23.96	15.58	2.33	10.98	20.19
R_AIP_ROI	40.89	5.06	30.90	50.89	12.30	1.72	8.91	15.69
L_AIP_ROI	36.20	4.05	28.19	44.22	4.10	0.61	2.88	5.31
R_EC_ROI	9.67	0.74	8.20	11.14	12.88	0.75	11.40	14.37
L_EC_ROI	11.99	0.70	10.60	13.37	13.37	0.91	11.56	15.18
R_PreS_ROI	17.60	1.23	15.16	20.03	15.86	1.42	13.05	18.67
L_PreS_ROI	16.42	1.16	14.12	18.72	17.61	1.19	15.25	19.97
R_H_ROI	22.71	1.42	19.91	25.52	37.60	2.32	33.01	42.18
L_H_ROI	24.38	1.76	20.91	27.85	43.94	2.45	39.10	48.78
R_ProS_ROI	14.14	1.40	11.38	16.90	13.08	1.69	9.74	16.43
L_ProS_ROI	14.36	1.34	11.70	17.01	14.50	1.65	11.24	17.76
R_PeEc_ROI	44.58	2.05	40.53	48.64	75.15	3.08	69.06	81.25
L_PeEc_ROI	41.37	1.86	37.68	45.05	71.04	2.83	65.44	76.63
R_STGa_ROI	24.86	1.10	22.69	27.03	33.25	0.82	31.62	34.88
L_STGa_ROI	36.45	1.10	34.27	38.63	43.11	1.02	41.08	45.13
R_PBelt_ROI	68.45	1.78	64.94	71.96	35.40	2.05	31.34	39.47
L_PBelt_ROI	114.13	2.99	108.23	120.03	52.87	3.03	46.88	58.86
R_A5_ROI	153.88	2.52	148.90	158.86	107.99	3.71	100.65	115.32
L_A5_ROI	104.95	2.29	100.42	109.49	88.55	2.44	83.73	93.37
R_PHA1_ROI	16.31	1.04	14.26	18.36	24.42	1.65	21.16	27.68
L_PHA1_ROI	13.35	1.12	11.13	15.56	25.33	1.53	22.31	28.36
R_PHA3_ROI	8.48	0.83	6.84	10.11	17.50	1.24	15.05	19.95
L_PHA3_ROI	11.50	0.97	9.58	13.42	29.58	1.99	25.65	33.51
R_STSda_ROI	76.05	3.24	69.65	82.45	142.12	2.52	137.14	147.10
L_STSda_ROI	64.86	2.20	60.50	69.22	100.48	1.91	96.70	104.25
R_STSdp_ROI	116.40	4.17	108.15	124.66	98.81	4.88	89.16	108.45
L_STSdp_ROI	143.80	3.32	137.23	150.37	134.17	3.46	127.33	141.00
R_STSvp_ROI	44.02	2.63	38.83	49.22	53.95	2.75	48.51	59.40
L_STSvp_ROI	90.62	4.37	81.97	99.27	119.14	4.32	110.61	127.68
R_TGd_ROI	105.04	4.29	96.55	113.52	225.85	5.27	215.43	236.26
L_TGd_ROI	118.98	5.67	107.76	130.19	256.10	5.90	244.42	267.77
R_TE1a_ROI	20.48	1.39	17.72	23.23	55.52	2.06	51.44	59.61
L_TE1a_ROI	30.51	2.08	26.39	34.63	82.19	2.33	77.58	86.80
R_TE1p_ROI	22.18	2.19	17.86	26.50	14.86	1.25	12.38	17.33
L_TE1p_ROI	35.11	2.94	29.29	40.93	41.55	2.61	36.39	46.71
R_TE2a_ROI	36.56	2.03	32.55	40.57	56.00	2.90	50.27	61.73
L_TE2a_ROI	36.63	2.00	32.67	40.59	51.79	2.25	47.33	56.24
R_TF_ROI	51.19	2.25	46.75	55.64	79.15	3.10	73.03	85.28
L_TF_ROI	65.71	2.42	60.93	70.50	109.32	3.49	102.42	116.23
R_TE2p_ROI	18.13	1.30	15.56	20.70	36.11	2.20	31.75	40.47
L_TE2p_ROI	21.77	1.24	19.32	24.23	30.25	1.79	26.72	33.78
R_PHT_ROI	9.11	1.05	7.04	11.18	18.36	1.55	15.29	21.43
L_PHT_ROI	22.21	1.93	18.40	26.03	37.86	2.40	33.11	42.61
R_PH_ROI	20.06	1.90	16.31	23.81	38.49	2.88	32.79	44.18
L_PH_ROI	21.86	2.54	16.84	26.87	35.70	2.55	30.66	40.74
R_TPOJ1_ROI	103.39	5.72	92.09	114.70	101.21	6.24	88.88	113.55
L_TPOJ1_ROI	82.15	4.25	73.75	90.56	70.80	3.79	63.31	78.29
R_TPOJ2_ROI	16.55	1.88	12.82	20.27	34.49	2.81	28.93	40.04
L_TPOJ2_ROI	22.56	2.28	18.06	27.06	40.68	2.93	34.88	46.48
R_TPOJ3_ROI	13.08	1.15	10.80	15.37	30.21	2.09	26.08	34.35
L_TPOJ3_ROI	10.93	1.22	8.52	13.34	27.74	1.74	24.30	31.18
R_DVT_ROI	5.55	0.79	3.99	7.11	11.83	1.44	8.99	14.68
L_DVT_ROI	5.62	0.73	4.18	7.06	11.00	1.20	8.62	13.38
R_PGp_ROI	10.36	1.32	7.75	12.96	27.68	2.56	22.61	32.75
L_PGp_ROI	6.73	0.95	4.86	8.59	20.85	1.96	16.97	24.72
R_IP2_ROI	5.80	1.19	3.45	8.14	0.00	0.00	0.00	0.00
L_IP2_ROI	6.63	1.18	4.29	8.97	0.00	0.00	0.00	0.00
R_IP1_ROI	7.58	1.42	4.78	10.38	0.61	0.28	0.05	1.17
L_IP1_ROI	9.71	1.59	6.57	12.86	1.38	0.40	0.59	2.18
R_IP0_ROI	4.93	0.73	3.48	6.38	4.54	0.77	3.00	6.07
L_IP0_ROI	6.06	0.84	4.41	7.71	4.24	0.85	2.56	5.91
R_PFop_ROI	9.18	1.13	6.95	11.41	29.74	2.85	24.11	35.36
L_PFop_ROI	11.76	1.35	9.08	14.44	23.42	2.57	18.34	28.49
R_PF_ROI	11.57	1.83	7.96	15.18	58.26	4.60	49.17	67.35
L_PF_ROI	21.45	2.49	16.53	26.37	36.88	3.43	30.09	43.67
R_PFm_ROI	21.68	2.35	17.03	26.33	13.87	1.94	10.04	17.70
L_PFm_ROI	36.87	3.75	29.46	44.28	51.88	3.46	45.04	58.73
R_PGi_ROI	96.29	7.86	80.75	111.82	233.69	7.51	218.83	248.55
L_PGi_ROI	152.98	8.54	136.09	169.86	337.48	6.08	325.45	349.50
R_PGs_ROI	9.75	1.47	6.84	12.66	22.70	2.05	18.64	26.76
L_PGs_ROI	23.54	2.44	18.72	28.35	99.89	4.27	91.46	108.33
R_V6A_ROI	1.58	0.33	0.93	2.24	9.43	1.14	7.17	11.69
L_V6A_ROI	1.43	0.29	0.86	2.00	7.37	1.00	5.40	9.34
R_VMV1_ROI	11.77	1.29	9.22	14.32	11.14	1.28	8.62	13.67
L_VMV1_ROI	7.57	0.93	5.72	9.42	5.94	0.90	4.15	7.73
R_VMV3_ROI	5.25	0.77	3.72	6.78	11.20	1.06	9.11	13.30
L_VMV3_ROI	4.70	0.62	3.48	5.92	8.81	1.21	6.41	11.21
R_PHA2_ROI	7.51	0.65	6.22	8.80	12.35	0.98	10.40	14.29
L_PHA2_ROI	7.74	0.72	6.31	9.17	20.10	1.31	17.51	22.68
R_V4t_ROI	6.20	0.71	4.79	7.61	19.46	1.32	16.85	22.07
L_V4t_ROI	2.81	0.45	1.91	3.71	14.74	1.00	12.75	16.72
R_FST_ROI	9.76	1.26	7.28	12.25	30.19	2.16	25.92	34.46
L_FST_ROI	8.46	1.31	5.87	11.06	27.39	1.93	23.58	31.20
R_V3CD_ROI	9.48	1.36	6.78	12.17	27.15	2.52	22.17	32.14
L_V3CD_ROI	4.81	0.83	3.17	6.45	18.67	1.78	15.14	22.19
R_LO3_ROI	4.46	0.63	3.22	5.71	18.44	1.76	14.95	21.93
L_LO3_ROI	2.38	0.40	1.58	3.18	11.79	1.46	8.89	14.68
R_VMV2_ROI	8.00	0.97	6.08	9.92	15.25	1.77	11.74	18.76
L_VMV2_ROI	3.38	0.55	2.29	4.48	5.39	0.88	3.65	7.14
R_31pd_ROI	4.93	0.94	3.07	6.79	46.83	2.34	42.20	51.47
L_31pd_ROI	15.56	1.53	12.53	18.59	76.45	2.66	71.19	81.72
R_31a_ROI	1.68	0.40	0.90	2.46	3.68	0.63	2.43	4.93
L_31a_ROI	1.63	0.36	0.93	2.33	8.26	1.06	6.17	10.35
R_VVC_ROI	13.40	1.10	11.24	15.57	26.17	1.77	22.67	29.66
L_VVC_ROI	11.96	1.00	9.98	13.95	26.50	1.77	23.01	29.99
R_25_ROI	10.40	0.69	9.04	11.77	19.48	0.94	17.63	21.33
L_25_ROI	9.37	0.60	8.19	10.55	17.00	0.85	15.32	18.68
R_s32_ROI	7.14	0.59	5.99	8.30	23.96	1.15	21.69	26.24
L_s32_ROI	7.96	0.72	6.55	9.38	26.98	1.11	24.78	29.17
R_pOFC_ROI	15.23	1.14	12.97	17.48	17.32	1.22	14.91	19.73
L_pOFC_ROI	8.00	0.61	6.79	9.21	11.23	0.87	9.51	12.94
R_PoI1_ROI	12.12	1.30	9.54	14.70	31.00	2.32	26.42	35.58
L_PoI1_ROI	11.49	1.36	8.79	14.19	28.13	2.28	23.63	32.63
R_Ig_ROI	12.95	1.27	10.45	15.45	29.19	2.45	24.34	34.04
L_Ig_ROI	5.98	0.70	4.58	7.37	9.55	1.02	7.53	11.56
R_FOP5_ROI	21.36	1.70	18.00	24.71	12.61	1.27	10.10	15.11
L_FOP5_ROI	22.33	1.80	18.76	25.90	8.83	0.91	7.03	10.63
R_p10p_ROI	7.99	0.80	6.40	9.58	18.50	1.61	15.32	21.68
L_p10p_ROI	3.74	0.77	2.22	5.25	9.96	1.04	7.90	12.03
R_p47r_ROI	5.96	0.83	4.33	7.60	7.38	0.91	5.59	9.17
L_p47r_ROI	16.19	1.75	12.73	19.65	14.00	1.36	11.31	16.69
R_TGv_ROI	23.60	1.16	21.31	25.88	42.10	1.88	38.39	45.80
L_TGv_ROI	19.75	1.25	17.28	22.22	31.11	1.73	27.68	34.53
R_MBelt_ROI	44.85	1.53	41.81	47.88	23.08	1.37	20.37	25.80
L_MBelt_ROI	55.12	1.91	51.35	58.89	28.19	1.84	24.55	31.83
R_LBelt_ROI	59.20	1.80	55.64	62.76	21.56	1.99	17.63	25.49
L_LBelt_ROI	57.07	1.73	53.65	60.50	19.56	1.67	16.27	22.85
R_A4_ROI	119.64	2.68	114.35	124.94	64.88	3.09	58.77	70.99
L_A4_ROI	138.24	2.95	132.41	144.07	90.32	2.99	84.42	96.23
R_STSva_ROI	21.29	1.56	18.20	24.38	57.12	1.37	54.41	59.83
L_STSva_ROI	57.95	2.74	52.53	63.38	114.06	1.54	111.01	117.11
R_TE1m_ROI	8.93	0.87	7.21	10.65	4.49	0.55	3.39	5.58
L_TE1m_ROI	9.61	1.25	7.14	12.07	12.10	1.01	10.09	14.10
R_PI_ROI	21.14	1.24	18.69	23.60	23.96	1.49	21.02	26.91
L_PI_ROI	26.05	1.49	23.10	29.00	27.60	1.87	23.90	31.29
R_a32pr_ROI	6.57	0.88	4.82	8.32	0.42	0.12	0.17	0.66
L_a32pr_ROI	6.77	1.04	4.72	8.82	0.80	0.24	0.33	1.27
R_p24_ROI	1.65	0.28	1.10	2.21	3.50	0.49	2.53	4.47
L_p24_ROI	1.10	0.23	0.63	1.56	2.21	0.34	1.54	2.89

The MMP area with the largest number of PEs was the right A5 complex (R_A5_ROI, located within Brodmann 22) in the STORY contrast with a mean value of 153.88 ± 2.52, and the left PGi (L_PGi_ROI, located within Brodmann 39 and 40), in the STORY-MATH with a mean value 337.48 ± 6.08.

Difference between the two hemispheres

**Figure 4:**
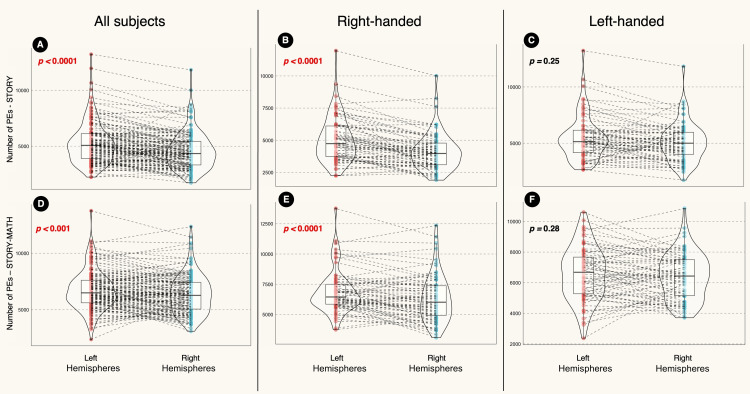
Violin plot showing the difference in the positive element (PE) numbers between the right and left hemispheres. The left hemisphere had significantly higher PEs than the right hemisphere in the STORY contrast for the whole sample (A) and RH group (B). Similarly, there were significant differences between the hemispheres in the entire sample (D) and RH group (E) in the STORY-MATH contrast. However, there were no significant differences between both hemispheres in the LH group (C and F). The results represent the outcome of the robust paired-sample t-test analyses. LH: Left-handed; RH: right-handed

In the STORY contrast, the left side of the brain had significantly higher numbers of PEs than the right. This was the case when analyzing the entire sample (n = 140, p < 0.0001, Figure 4A) or the RH subgroup (n = 70, p < 0.0001, Figure 4B). The exception was in the LH subgroup that showed no differences between both hemispheres (p = 0.25, Figure 4C).

Also, the STORY-MATH contrast showed a significant difference between the two sides of the brain, with the left side of the brain having significantly higher numbers of PEs than the right when analyzing the entire sample (n =140, p < 0.001, Figure 4D). The same finding was seen in the RH subgroup (n = 70, p < 0.0001, Figure 4E). Again, the exception was in the LH subgroup that showed no differences between both hemispheres (n = 70, p = 0.28, Figure 4F).

Language dominance in the MMP regions

Entire Sample (n = 140)

**Figure 5:**
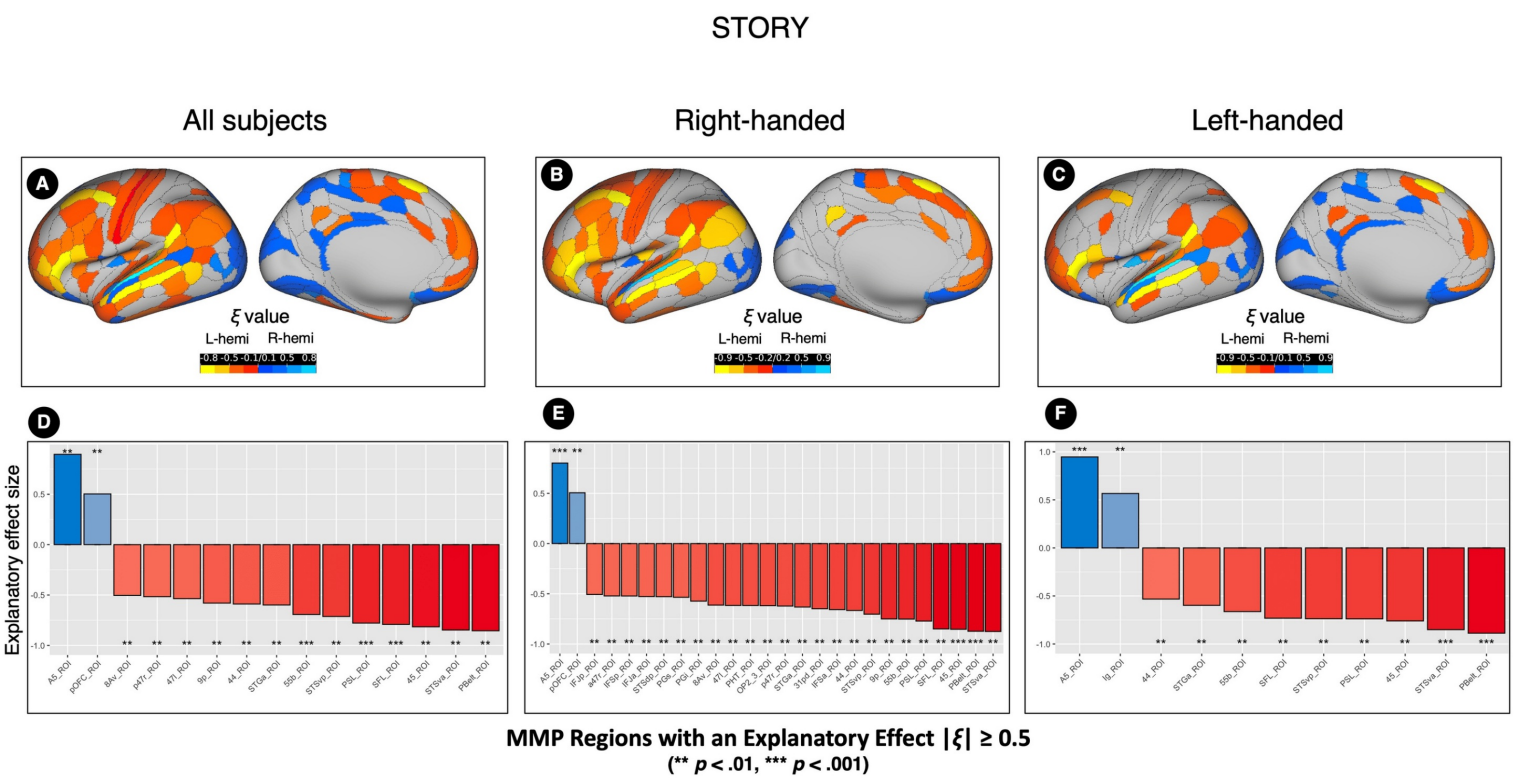
The explanatory measure of the effect size (ξ) values of the mean difference in the positive element (PE) numbers between the right and left sides of each MMP region in the STORY contrast The upper row displays ξ values mapped onto an inflated hemispheric template for the entire sample (n=140, A), right-handed participants (n=70, B), and left-handed participants (n=70, C). The brain surface template serves as a spatial reference and does not represent the anatomical left hemisphere. Yellow regions indicate significantly more PEs in the left hemisphere, while blue regions indicate significantly more PEs in the right hemisphere. The lower row shows bar charts for regions with large ξ values across the entire sample (n=140, D), right-handed group (n=70, E), and left-handed group (n=70, F). Red bars denote regions with significantly more PEs in the left hemisphere, and blue bars denote those with significantly more PEs in the right hemisphere. ξ values and p-values were calculated using robust paired-sample t-test analyses. L-hemi: Left hemisphere; R-hemi: right hemisphere; MMP: multimodal parcellation

In the STORY contrast, 103 MMP regions showed significant differences (corrected p values) in the PE number between the hemispheres (Table 5, Figure 5A). Regions with an absolute ξ value equal to or greater than 0.5 are shown in Figure 5D. The area with significantly more PEs in the left hemisphere and expressing the largest absolute ξ value was the ParaBelt complex (PBelt_ROI, located within Brodmann 22 and 42), with a ξ = 0.9 and a mean difference value of 45.68 (CI 95% 40.16 ~ 51.2). The area with significantly more PEs in the right hemisphere and expressing the largest absolute ξ value was the A5 complex (A5_ROI, located within Brodmann 22), with a ξ = 0.9 and a mean difference value of 48.93 (CI 95% 43.60 ~ 54.25).

**Table 5:** The difference in the positive element (PE) numbers in each MMP region between the two hemispheres in the STORY contrast. Data are presented in descending order of the explanatory measure of effect size (ξ). Results with a positive effect size are in the direction of the right hemisphere, while negative results are in the direction of the left hemisphere. Only regions with a corrected p ≤ 0.05 are shown. The results were analyzed using the robust paired-sample t-test. MMP: Multimodal parcellation

	MMP Region	Statistics	Mean Difference	95% CI		p	p (corrected)	ξ
				Lower	Upper			
1	A5_ROI	18.27	48.93	43.60	54.25	1E-16	0.0017	0.9
2	pOFC_ROI	5.68	7.23	4.69	9.76	1.97029E-07	0.0072	0.5
3	Ig_ROI	5.25	6.98	4.33	9.62	1.14421E-06	0.0100	0.5
4	VMV2_ROI	5.55	4.62	2.96	6.27	3.34975E-07	0.0083	0.4
5	TA2_ROI	5.11	9.46	5.78	13.15	1.99902E-06	0.0103	0.4
6	V4t_ROI	4.89	3.39	2.01	4.77	4.92287E-06	0.0114	0.4
7	5m_ROI	5.45	6.54	4.15	8.92	5.04781E-07	0.0086	0.4
8	V8_ROI	5.70	3.62	2.36	4.88	1.74761E-07	0.0069	0.3
9	MST_ROI	5.32	4.48	2.80	6.15	8.57659E-07	0.0097	0.3
10	p10p_ROI	4.82	4.25	2.50	6.00	6.31851E-06	0.0122	0.3
11	LO1_ROI	4.45	2.82	1.56	4.08	2.61015E-05	0.0136	0.3
12	TPOJ1_ROI	3.39	21.24	8.77	33.70	0.001074653	0.0189	0.3
13	V3_ROI	8.20	17.30	13.10	21.50	2.6499E-12	0.0031	0.3
14	PIT_ROI	4.36	3.49	1.90	5.08	3.75218E-05	0.0142	0.3
15	OFC_ROI	4.03	8.69	4.40	12.98	0.000124264	0.0161	0.3
16	s6-8_ROI	3.96	2.21	1.10	3.33	0.000156525	0.0167	0.3
17	STSda_ROI	3.42	11.19	4.68	17.70	0.000970876	0.0186	0.3
18	V3CD_ROI	4.62	4.67	2.66	6.67	1.36918E-05	0.0131	0.3
19	FOP1_ROI	3.97	2.23	1.11	3.34	0.000152234	0.0164	0.3
20	LO3_ROI	4.26	2.08	1.11	3.06	5.28215E-05	0.0150	0.2
21	VMV1_ROI	3.78	4.20	1.99	6.41	0.000296413	0.0169	0.2
22	v23ab_ROI	2.86	2.33	0.71	3.96	0.005428181	0.0228	0.2
23	PGp_ROI	3.24	3.63	1.40	5.86	0.001705586	0.0200	0.2
24	5mv_ROI	3.44	5.00	2.11	7.89	0.000911437	0.0183	0.2
25	47s_ROI	3.17	4.85	1.81	7.88	0.002118642	0.0206	0.2
26	TGv_ROI	2.53	3.85	0.82	6.87	0.013312431	0.0258	0.2
27	V7_ROI	2.55	1.27	0.28	2.27	0.012675898	0.0253	0.2
28	23d_ROI	2.38	1.25	0.21	2.29	0.019575638	0.0275	0.2
29	PHA1_ROI	2.27	2.96	0.37	5.56	0.025712066	0.0283	0.2
30	11l_ROI	2.47	2.69	0.52	4.86	0.01551734	0.0261	0.2
31	RSC_ROI	2.55	2.10	0.46	3.73	0.012484828	0.0250	0.2
32	FFC_ROI	2.41	4.05	0.71	7.39	0.018099198	0.0267	0.2
33	MIP_ROI	2.71	3.98	1.06	6.89	0.008068986	0.0244	0.2
34	24dv_ROI	2.26	3.15	0.38	5.93	0.026194829	0.0286	0.2
35	7Pm_ROI	2.35	2.42	0.37	4.46	0.021254825	0.0278	0.1
36	7Am_ROI	2.74	3.30	0.90	5.69	0.007521125	0.0236	0.1
37	V4_ROI	2.93	8.90	2.86	14.95	0.004375608	0.0219	0.1
38	V2_ROI	3.75	11.65	5.48	17.83	0.000324659	0.0178	0.1
39	V3A_ROI	2.72	2.74	0.74	4.74	0.007922004	0.0242	0.1
40	3a_ROI	-2.39	-6.40	-11.72	-1.09	0.018874492	0.0272	-0.1
41	FEF_ROI	-2.28	-4.95	-9.27	-0.64	0.025019612	0.0281	-0.2
42	4_ROI	-3.02	-16.71	-27.74	-5.69	0.003406085	0.0217	-0.2
43	LIPd_ROI	-2.87	-2.42	-4.09	-0.74	0.005209291	0.0225	-0.2
44	TGd_ROI	-2.44	-13.94	-25.32	-2.56	0.016998911	0.0264	-0.2
45	TE2p_ROI	-2.41	-3.64	-6.65	-0.63	0.018339876	0.0269	-0.2
46	3b_ROI	-3.06	-13.11	-21.63	-4.58	0.003004572	0.0211	-0.2
47	24dd_ROI	-2.81	-6.58	-11.25	-1.92	0.006197139	0.0231	-0.2
48	TPOJ2_ROI	-2.54	-6.01	-10.71	-1.31	0.012834903	0.0256	-0.2
49	EC_ROI	-2.68	-2.32	-4.05	-0.60	0.008963599	0.0247	-0.2
50	PHA3_ROI	-2.73	-3.02	-5.22	-0.82	0.007636809	0.0239	-0.2
51	8C_ROI	-2.78	-12.40	-21.28	-3.53	0.006736037	0.0233	-0.2
52	6r_ROI	-3.76	-8.67	-13.25	-4.08	0.000317284	0.0172	-0.2
53	10d_ROI	-3.20	-3.45	-5.60	-1.31	0.001928225	0.0203	-0.2
54	MI_ROI	-3.27	-2.57	-4.13	-1.01	0.001547266	0.0197	-0.2
55	1_ROI	-3.12	-13.04	-21.34	-4.73	0.002475508	0.0208	-0.2
56	PI_ROI	-2.92	-4.90	-8.25	-1.56	0.004556645	0.0222	-0.2
57	SCEF_ROI	-3.75	-9.29	-14.21	-4.37	0.000322271	0.0175	-0.2
58	a9-46v_ROI	-3.37	-2.52	-4.01	-1.04	0.00113421	0.0192	-0.2
59	a24pr_ROI	-3.05	-2.21	-3.66	-0.77	0.00304903	0.0214	-0.3
60	d32_ROI	-3.61	-3.01	-4.67	-1.35	0.000516353	0.0181	-0.3
61	52_ROI	-3.36	-4.45	-7.09	-1.82	0.001167515	0.0194	-0.3
62	PF_ROI	-4.10	-9.88	-14.68	-5.09	9.64591E-05	0.0156	-0.3
63	PFm_ROI	-4.26	-15.19	-22.29	-8.09	5.44782E-05	0.0153	-0.3
64	10r_ROI	-4.07	-3.04	-4.52	-1.55	0.00010659	0.0158	-0.3
65	A1_ROI	-4.36	-6.81	-9.92	-3.70	3.69362E-05	0.0139	-0.3
66	9-46d_ROI	-4.84	-6.98	-9.85	-4.11	6.00687E-06	0.0119	-0.3
67	TE1p_ROI	-5.04	-12.93	-18.04	-7.82	2.73075E-06	0.0106	-0.3
68	IFSp_ROI	-4.49	-11.69	-16.87	-6.51	2.25527E-05	0.0133	-0.3
69	IFJa_ROI	-4.67	-10.73	-15.29	-6.16	1.13965E-05	0.0128	-0.3
70	d23ab_ROI	-4.27	-2.23	-3.26	-1.19	5.21519E-05	0.0147	-0.3
71	a10p_ROI	-4.29	-4.15	-6.08	-2.23	4.74275E-05	0.0144	-0.4
72	TE1a_ROI	-5.58	-10.04	-13.61	-6.46	2.89209E-07	0.0078	-0.4
73	MBelt_ROI	-5.44	-10.27	-14.03	-6.52	5.2806E-07	0.0089	-0.4
74	IFJp_ROI	-5.00	-7.05	-9.85	-4.25	3.10253E-06	0.0108	-0.4
75	FOP4_ROI	-4.87	-8.96	-12.63	-5.30	5.32085E-06	0.0117	-0.4
76	TF_ROI	-4.75	-14.52	-20.60	-8.45	8.28591E-06	0.0125	-0.4
77	STSdp_ROI	-5.65	-27.39	-37.04	-17.75	2.21033E-07	0.0075	-0.4
78	a47r_ROI	-5.40	-10.54	-14.41	-6.66	6.11926E-07	0.0092	-0.4
79	9m_ROI	-6.79	-17.77	-22.98	-12.57	1.58091E-09	0.0039	-0.4
80	OP1_ROI	-4.89	-9.31	-13.09	-5.53	4.81105E-06	0.0111	-0.4
81	10v_ROI	-6.06	-7.57	-10.06	-5.09	3.80166E-08	0.0061	-0.4
82	STV_ROI	-5.33	-14.37	-19.73	-9.01	8.31346E-07	0.0094	-0.4
83	A4_ROI	-5.83	-18.60	-24.94	-12.25	1.0398E-07	0.0067	-0.4
84	PGs_ROI	-5.90	-13.79	-18.43	-9.14	7.51652E-08	0.0064	-0.4
85	PGi_ROI	-6.67	-56.69	-73.58	-39.80	2.61067E-09	0.0042	-0.4
86	9a_ROI	-6.43	-10.68	-13.98	-7.38	7.53199E-09	0.0050	-0.4
87	OP2-3_ROI	-6.08	-9.12	-12.10	-6.14	3.4625E-08	0.0058	-0.5
88	31pd_ROI	-6.47	-10.63	-13.90	-7.36	6.34135E-09	0.0047	-0.5
89	IFSa_ROI	-5.56	-9.39	-12.75	-6.03	3.23817E-07	0.0081	-0.5
90	PHT_ROI	-6.86	-13.11	-16.91	-9.31	1.14076E-09	0.0036	-0.5
91	8Av_ROI	-6.57	-18.64	-24.28	-13.00	4.0956E-09	0.0044	-0.5
92	p47r_ROI	-6.37	-10.23	-13.42	-7.03	1.00643E-08	0.0053	-0.5
93	47l_ROI	-8.82	-19.88	-24.37	-15.40	1.53E-13	0.0028	-0.5
94	9p_ROI	-6.22	-10.76	-14.20	-7.32	1.88129E-08	0.0056	-0.6
95	44_ROI	-7.70	-27.99	-35.22	-20.76	2.59268E-11	0.0033	-0.6
96	STGa_ROI	-9.04	-11.60	-14.15	-9.04	5.37E-14	0.0025	-0.6
97	55b_ROI	-10.37	-35.73	-42.58	-28.87	1E-16	0.0003	-0.7
98	STSvp_ROI	-10.73	-46.60	-55.24	-37.96	1E-16	0.0019	-0.7
99	PSL_ROI	-11.59	-53.64	-62.85	-44.44	1E-16	0.0006	-0.8
100	SFL_ROI	-11.37	-38.46	-45.19	-31.74	1E-16	0.0008	-0.8
101	45_ROI	-13.55	-51.93	-59.55	-44.31	1E-16	0.0011	-0.8
102	STSva_ROI	-13.58	-36.67	-42.04	-31.30	1E-16	0.0022	-0.8
103	PBelt_ROI	-16.47	-45.68	-51.20	-40.16	1E-16	0.0014	-0.9

**Figure 6:**
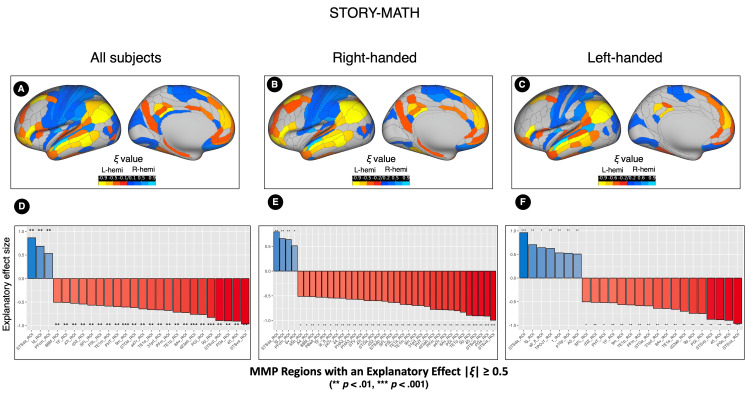
The explanatory measure of the effect size (ξ) values of the mean difference in the positive element (PE) numbers between the right and left sides of each MMP region in the STORY-MATH contrast. The upper row displays ξ values mapped onto an inflated hemispheric template for the entire sample (n=140, A), right-handed participants (n=70, B), and left-handed participants (n=70, C). The brain surface template serves as a spatial reference and does not represent the anatomical left hemisphere. Yellow regions indicate significantly more PEs in the left hemisphere, while blue regions indicate significantly more PEs in the right hemisphere. The lower row shows bar charts for regions with large ξ values across the entire sample (n=140, D), right-handed group (n=70, E), and left-handed group (n=70, F). Red bars denote regions with significantly more PEs in the left hemisphere, and blue bars denote those with significantly more PEs in the right hemisphere. ξ values and p-values were calculated using robust paired-sample t-test analyses. L-hemi: Left hemisphere; R-hemi: right hemisphere; MMP: multimodal parcellation

In the STORY-MATH contrast, 116 MMP regions showed significant differences (corrected p values) in the PE number between the hemispheres (Table 6, Figure 6A). Regions with an absolute ξ value equal to or greater than 0.5 are shown in Figure 6D. The area with significantly more PEs in the left hemisphere and expressing the largest absolute ξ value was the Area STSv anterior (STSva_ROI, located within Brodmann 22), with a ξ = 1 and a mean difference value of 56.94 (CI 95% 53.35 ~ 60.53). The area with significantly more PEs in the right hemisphere and expressing the largest absolute ξ value was the STSd anterior (STSda_ROI, located within Brodmann 22), with a ξ = 0.9 and a mean difference value of 41.64 (CI 95% 36.1 ~ 47.18). For more information about the regions' location and name description, please refer to Figure 1.

**Table 6:** The difference in the positive element (PE) numbers in each MMP region between the two hemispheres in the STORY-MATH contrast. Data are presented in descending order of the explanatory measure of effect size (ξ). Results with positive effect size are in the direction of the right hemisphere, while negative results are in the direction of the left hemisphere. Only regions with a corrected p ≤ 0.05 are shown. The results were analyzed using the robust paired-sample t-test. MMP: Multimodal parcellation

	MMP Region	Statistics	Mean Difference	95% CI		p	p (corrected)	ξ
				Lower	Upper			
1	STSda_ROI	14.9493	41.64	36.10	47.1833	1E-16	0.0014	0.9
2	Ig_ROI	8.47	19.64	15.03	24.26	7.55E-13	0.0050	0.7
3	PFcm_ROI	7.49	29.83	21.91	37.76	6.8322E-11	0.0058	0.5
4	1_ROI	8.55	40.45	31.04	49.87	5.251E-13	0.0047	0.5
5	VMV2_ROI	6.48	9.86	6.83	12.88	6.29063E-09	0.0092	0.5
6	s6-8_ROI	4.70	4.04	2.33	5.74	1.00302E-05	0.0161	0.5
7	6v_ROI	5.96	7.14	4.76	9.53	5.9589E-08	0.0111	0.4
8	TPOJ1_ROI	4.38	30.42	16.61	44.23	3.4358E-05	0.0178	0.4
9	LIPv_ROI	6.05	9.79	6.57	13.00	3.96309E-08	0.0108	0.4
10	p10p_ROI	4.99	8.54	5.13	11.94	3.2759E-06	0.0153	0.4
11	A5_ROI	5.02	19.44	11.74	27.14	2.89386E-06	0.0150	0.4
12	AIP_ROI	5.36	8.20	5.16	11.25	7.33678E-07	0.0133	0.4
13	TGv_ROI	5.25	10.99	6.83	15.15	1.14099E-06	0.0142	0.4
14	OP4_ROI	5.55	20.96	13.45	28.48	3.31916E-07	0.0122	0.4
15	OFC_ROI	5.31	17.00	10.63	23.37	9.09802E-07	0.0136	0.4
16	2_ROI	6.37	41.40	28.48	54.33	9.83543E-09	0.0097	0.4
17	pOFC_ROI	4.56	6.10	3.44	8.76	1.76051E-05	0.0169	0.4
18	PF_ROI	4.87	21.38	12.64	30.12	5.31025E-06	0.0156	0.3
19	5m_ROI	5.70	11.75	7.65	15.85	1.8032E-07	0.0119	0.3
20	6d_ROI	5.05	9.08	5.51	12.66	2.53865E-06	0.0147	0.3
21	v23ab_ROI	5.71	9.71	6.33	13.10	1.67761E-07	0.0117	0.3
22	24dd_ROI	4.58	13.10	7.41	18.79	1.63624E-05	0.0167	0.3
23	V3_ROI	7.10	21.93	15.78	28.07	3.95387E-10	0.0067	0.3
24	RI_ROI	3.84	14.96	7.22	22.71	0.000236496	0.0217	0.3
25	FOP2_ROI	3.99	5.87	2.94	8.79	0.000141535	0.0211	0.3
26	7PC_ROI	4.34	10.04	5.43	14.64	4.05909E-05	0.0183	0.3
27	43_ROI	4.37	10.25	5.58	14.92	3.60819E-05	0.0181	0.3
28	VMV1_ROI	4.43	5.20	2.87	7.54	2.81439E-05	0.0175	0.3
29	MST_ROI	4.03	7.86	3.98	11.73	0.000121683	0.0206	0.3
30	TA2_ROI	3.67	6.01	2.75	9.27	0.000432824	0.0233	0.3
31	LO3_ROI	4.33	6.65	3.60	9.71	4.15857E-05	0.0186	0.3
32	V3CD_ROI	4.76	8.49	4.94	12.04	8.20356E-06	0.0158	0.3
33	V4t_ROI	3.71	4.73	2.19	7.26	0.00037165	0.0231	0.2
34	LO1_ROI	4.16	4.86	2.53	7.18	7.75411E-05	0.0200	0.2
35	3a_ROI	5.51	17.69	11.30	24.08	4.02105E-07	0.0125	0.2
36	4_ROI	5.21	36.45	22.55	50.36	1.33334E-06	0.0144	0.2
37	PFt_ROI	3.64	9.63	4.37	14.89	0.000471588	0.0236	0.2
38	VIP_ROI	4.00	4.76	2.39	7.13	0.000137843	0.0208	0.2
39	PoI2_ROI	2.94	6.82	2.20	11.44	0.004305374	0.0264	0.2
40	FOP5_ROI	2.93	3.77	1.21	6.34	0.004371874	0.0267	0.2
41	p24_ROI	2.60	1.29	0.30	2.27	0.011164025	0.0278	0.2
42	V8_ROI	3.17	3.81	1.42	6.20	0.002111053	0.0253	0.2
43	PEF_ROI	2.53	0.85	0.18	1.51	0.013366006	0.0286	0.2
44	6mp_ROI	2.94	9.62	3.11	16.13	0.004269059	0.0261	0.2
45	3b_ROI	3.77	23.32	11.01	35.64	0.00030847	0.0222	0.2
46	TE2p_ROI	2.23	5.86	0.63	11.09	0.028616851	0.0319	0.2
47	FOP1_ROI	3.12	2.37	0.86	3.88	0.002485105	0.0258	0.2
48	RSC_ROI	2.54	4.19	0.91	7.47	0.012963853	0.0283	0.2
49	PGp_ROI	3.40	6.83	2.83	10.84	0.001053272	0.0247	0.2
50	6r_ROI	2.24	3.23	0.37	6.09	0.02757558	0.0311	0.2
51	MIP_ROI	2.46	1.26	0.24	2.28	0.016056484	0.0292	0.2
52	25_ROI	2.32	2.48	0.35	4.60	0.022865034	0.0306	0.2
53	FEF_ROI	2.24	2.60	0.29	4.90	0.027746419	0.0314	0.2
54	SCEF_ROI	2.43	1.82	0.33	3.31	0.017148527	0.0294	0.2
55	V7_ROI	2.23	2.13	0.23	4.03	0.028231215	0.0317	0.2
56	PFop_ROI	2.39	6.32	1.06	11.58	0.019035833	0.0297	0.1
57	PIT_ROI	2.39	3.07	0.51	5.63	0.019180388	0.0300	0.1
58	VMV3_ROI	2.21	2.39	0.24	4.54	0.029556335	0.0322	0.1
59	V2_ROI	3.24	10.12	3.90	16.34	0.001741972	0.0250	0.1
60	V6_ROI	2.77	3.29	0.93	5.64	0.006868286	0.0269	0.1
61	V3A_ROI	2.31	4.46	0.62	8.31	0.023472358	0.0308	0.1
62	H_ROI	-2.49	-6.35	-11.42	-1.28	0.014804367	0.0289	-0.2
63	LO2_ROI	-2.62	-3.33	-5.86	-0.81	0.010385084	0.0275	-0.2
64	s32_ROI	-2.38	-3.01	-5.52	-0.50	0.019383197	0.0303	-0.2
65	POS1_ROI	-3.45	-8.71	-13.74	-3.69	0.000885439	0.0242	-0.2
66	MBelt_ROI	-2.68	-5.11	-8.89	-1.32	0.008807041	0.0272	-0.2
67	11l_ROI	-2.59	-3.81	-6.73	-0.89	0.011305997	0.0281	-0.2
68	MT_ROI	-3.12	-4.14	-6.78	-1.50	0.002481512	0.0256	-0.2
69	POS2_ROI	-4.23	-5.48	-8.05	-2.90	5.87884E-05	0.0194	-0.2
70	a24_ROI	-3.91	-4.21	-6.36	-2.07	0.000189147	0.0214	-0.2
71	FOP4_ROI	-3.56	-2.10	-3.27	-0.92	0.000623624	0.0239	-0.2
72	p24pr_ROI	-3.74	-4.93	-7.55	-2.31	0.000337583	0.0225	-0.3
73	31pv_ROI	-3.72	-11.76	-18.06	-5.47	0.000365869	0.0228	-0.3
74	46_ROI	-4.32	-4.79	-6.99	-2.58	4.37793E-05	0.0189	-0.3
75	i6-8_ROI	-3.40	-1.85	-2.93	-0.76	0.001048703	0.0244	-0.3
76	52_ROI	-4.05	-4.61	-6.87	-2.35	0.000113899	0.0203	-0.3
77	10r_ROI	-4.53	-7.25	-10.44	-4.06	1.9789E-05	0.0172	-0.3
78	31a_ROI	-4.23	-4.58	-6.74	-2.43	5.94233E-05	0.0197	-0.3
79	TGd_ROI	-5.47	-30.25	-41.25	-19.25	4.6276E-07	0.0131	-0.3
80	a24pr_ROI	-3.83	-3.76	-5.72	-1.81	0.000247085	0.0219	-0.3
81	p47r_ROI	-4.70	-6.62	-9.42	-3.82	1.03373E-05	0.0164	-0.4
82	10d_ROI	-6.10	-14.61	-19.37	-9.85	3.21058E-08	0.0106	-0.4
83	a10p_ROI	-4.25	-6.86	-10.07	-3.65	5.54498E-05	0.0192	-0.4
84	10v_ROI	-6.99	-12.26	-15.75	-8.77	6.38508E-10	0.0072	-0.4
85	PHA2_ROI	-5.50	-7.75	-10.55	-4.95	4.16972E-07	0.0128	-0.4
86	55b_ROI	-5.80	-13.89	-18.66	-9.13	1.18667E-07	0.0114	-0.4
87	44_ROI	-5.29	-14.45	-19.89	-9.01	9.94255E-07	0.0139	-0.4
88	PBelt_ROI	-6.27	-17.46	-23.01	-11.92	1.57344E-08	0.0103	-0.4
89	STV_ROI	-6.36	-18.75	-24.61	-12.89	1.04647E-08	0.0100	-0.5
90	A4_ROI	-7.33	-25.44	-32.35	-18.54	1.38981E-10	0.0061	-0.5
91	PHA3_ROI	-6.43	-12.08	-15.82	-8.35	7.60384E-09	0.0094	-0.5
92	9a_ROI	-7.85	-22.38	-28.05	-16.71	1.29556E-11	0.0053	-0.5
93	STSdp_ROI	-6.54	-35.36	-46.11	-24.60	4.77326E-09	0.0089	-0.5
94	8BM_ROI	-6.58	-10.31	-13.42	-7.19	3.92082E-09	0.0086	-0.5
95	TF_ROI	-7.27	-30.17	-38.42	-21.91	1.82952E-10	0.0064	-0.5
96	47l_ROI	-6.98	-18.01	-23.14	-12.88	6.5401E-10	0.0075	-0.5
97	d32_ROI	-6.97	-7.99	-10.27	-5.71	6.84006E-10	0.0078	-0.5
98	SFL_ROI	-6.80	-19.74	-25.51	-13.96	1.49461E-09	0.0081	-0.6
99	PSL_ROI	-7.00	-21.92	-28.15	-15.69	6.18909E-10	0.0069	-0.6
100	TE1m_ROI	-6.75	-7.61	-9.85	-5.36	1.90244E-09	0.0083	-0.6
101	PHT_ROI	-7.53	-19.50	-24.65	-14.35	5.4456E-11	0.0056	-0.6
102	9m_ROI	-12.94	-43.99	-50.75	-37.23	1E-16	0.0006	-0.6
103	STGa_ROI	-9.55	-9.86	-11.91	-7.80	5.3E-15	0.0042	-0.6
104	a47r_ROI	-9.46	-25.89	-31.34	-20.45	7.8E-15	0.0044	-0.6
105	TE1a_ROI	-11.51	-26.67	-31.27	-22.06	1E-16	0.0019	-0.7
106	31pd_ROI	-10.34	-29.62	-35.32	-23.92	1E-16	0.0028	-0.7
107	PFm_ROI	-10.10	-38.01	-45.50	-30.53	4E-16	0.0039	-0.7
108	TE1p_ROI	-10.07	-26.69	-31.96	-21.42	4E-16	0.0036	-0.7
109	8Av_ROI	-10.12	-44.32	-53.03	-35.61	4E-16	0.0033	-0.7
110	d23ab_ROI	-14.45	-17.71	-20.15	-15.28	1E-16	0.0003	-0.8
111	PGi_ROI	-12.54	-103.79	-120.25	-87.32	1E-16	0.0022	-0.8
112	9p_ROI	-13.61	-39.54	-45.32	-33.76	1E-16	0.0008	-0.8
113	STSvp_ROI	-14.61	-65.19	-74.07	-56.31	1E-16	0.0017	-0.9
114	PGs_ROI	-18.11	-77.19	-85.67	-68.71	1E-16	0.0025	-0.9
115	45_ROI	-18.32	-54.13	-60.01	-48.25	1E-16	0.0011	-0.9
116	STSva_ROI	-31.54	-56.94	-60.53	-53.35	1E-16	0.0031	-1.0

Right-Handed Group (n = 70)

In the STORY contrast, 83 MMP regions showed significant differences (corrected p values) in the PE number between the hemispheres (Table 7, Figure 5B). Regions with an absolute ξ value equal to or greater than 0.5 are shown in Figure 5E. The area with significantly more PEs in the left hemisphere and expressing the largest absolute ξ value was the Area STSv anterior (STSva_ROI, located within Brodmann 22), with a ξ = 0.9 and a mean difference value of 37.12 (CI 95% 29.47 ~ 44.77). The area with significantly more PEs in the right hemisphere and expressing the largest absolute ξ value was the A5 complex (A5_ROI, located within Brodmann 22), with a ξ = 0.8 and a mean difference value of 41.5 (CI 95% 34.02 ~ 48.98).

**Table 7:** The difference in the positive element (PE) numbers in each MMP region between the two hemispheres in the STORY contrast in the right-handed subjects only. Data are presented in descending order of the explanatory measure of effect size (ξ). Results with positive effect size are in the direction of the right hemisphere, while negative results are in the direction of the left hemisphere. Only regions with a corrected p ≤ 0.05 are shown. The results were analyzed using the robust paired-sample t-test. MMP: Multimodal parcellation

	MMP Region	Statistics	Mean Difference	95% CI		p	p (corrected)	ξ
				Lower	Upper			
1	A5_ROI	11.20	41.50	34.02	48.98	4.75E-14	0.0008	0.8
2	pOFC_ROI	4.41	7.14	3.87	10.41	7.32722E-05	0.0092	0.5
3	VMV2_ROI	3.44	4.31	1.78	6.84	0.001340968	0.0136	0.4
4	V8_ROI	4.42	3.40	1.85	4.96	7.19022E-05	0.0089	0.4
5	5m_ROI	3.08	5.62	1.94	9.30	0.003668321	0.0172	0.4
6	MST_ROI	3.75	4.38	2.02	6.74	0.000544171	0.0122	0.3
7	V3_ROI	6.38	17.12	11.70	22.54	1.25614E-07	0.0033	0.3
8	TA2_ROI	3.21	8.43	3.13	13.72	0.002548581	0.0153	0.3
9	Ig_ROI	2.64	4.62	1.08	8.16	0.011759355	0.0197	0.3
10	V3CD_ROI	2.97	4.43	1.42	7.44	0.0049838	0.0181	0.3
11	LO1_ROI	3.14	2.43	0.87	3.99	0.003099512	0.0158	0.3
12	LO3_ROI	3.11	1.74	0.61	2.87	0.003436035	0.0164	0.3
13	V4t_ROI	2.36	1.93	0.28	3.58	0.022853585	0.0231	0.3
14	PIT_ROI	2.41	2.52	0.41	4.64	0.020555726	0.0225	0.3
15	OFC_ROI	2.72	7.76	1.99	13.53	0.009597543	0.0192	0.3
16	VMV1_ROI	3.09	4.90	1.70	8.11	0.003581059	0.0169	0.3
17	V4_ROI	3.37	12.93	5.19	20.67	0.001627311	0.0139	0.2
18	s6_8_ROI	2.57	1.86	0.40	3.32	0.013817057	0.0208	0.2
19	MIP_ROI	2.54	5.07	1.03	9.11	0.015107786	0.0211	0.2
20	V3A_ROI	2.52	3.10	0.61	5.58	0.015722217	0.0214	0.2
21	6a_ROI	-2.71	-12.14	-21.20	-3.08	0.009864218	0.0194	-0.2
22	3a_ROI	-2.79	-9.57	-16.51	-2.64	0.008012114	0.0186	-0.2
23	3b_ROI	-2.96	-16.00	-26.90	-5.10	0.005028882	0.0183	-0.2
24	4_ROI	-2.76	-21.60	-37.37	-5.82	0.008507716	0.0189	-0.2
25	FEF_ROI	-2.48	-7.33	-13.30	-1.37	0.017195561	0.0219	-0.3
26	PHA3_ROI	-2.59	-3.83	-6.82	-0.84	0.013297822	0.0206	-0.3
27	8BM_ROI	-2.64	-8.57	-15.14	-2.00	0.011808232	0.0200	-0.3
28	9_46d_ROI	-3.05	-6.76	-11.25	-2.28	0.00404251	0.0175	-0.3
29	a9_46v_ROI	-2.50	-3.24	-5.86	-0.62	0.016695743	0.0217	-0.3
30	i6_8_ROI	-3.22	-4.36	-7.09	-1.63	0.002486812	0.0150	-0.3
31	SCEF_ROI	-2.60	-9.43	-16.74	-2.12	0.012750309	0.0203	-0.3
32	A1_ROI	-3.09	-7.29	-12.04	-2.53	0.003569099	0.0167	-0.3
33	PI_ROI	-2.38	-6.07	-11.22	-0.92	0.021971167	0.0228	-0.3
34	PF_ROI	-3.28	-9.12	-14.74	-3.50	0.002132962	0.0147	-0.3
35	10d_ROI	-3.45	-3.93	-6.23	-1.63	0.001295449	0.0133	-0.3
36	MI_ROI	-3.65	-4.14	-6.44	-1.85	0.000743617	0.0125	-0.3
37	52_ROI	-3.11	-5.07	-8.37	-1.77	0.003435171	0.0161	-0.3
38	TGd_ROI	-2.99	-22.76	-38.15	-7.38	0.004726247	0.0178	-0.3
39	PFm_ROI	-4.13	-16.95	-25.24	-8.67	0.000173057	0.0108	-0.3
40	AAIC_ROI	-2.44	-2.69	-4.92	-0.46	0.019122408	0.0222	-0.3
41	d23ab_ROI	-3.17	-1.88	-3.08	-0.68	0.002848123	0.0156	-0.3
42	6r_ROI	-4.06	-12.10	-18.11	-6.08	0.000213536	0.0111	-0.4
43	10r_ROI	-3.32	-3.69	-5.94	-1.45	0.001894189	0.0144	-0.4
44	a24pr_ROI	-3.37	-3.31	-5.29	-1.33	0.001646659	0.0142	-0.4
45	24dd_ROI	-4.26	-13.50	-19.90	-7.10	0.000116894	0.0103	-0.4
46	FOP4_ROI	-3.60	-9.57	-14.94	-4.20	0.000845446	0.0128	-0.4
47	8Ad_ROI	-4.32	-7.36	-10.80	-3.92	9.73117E-05	0.0100	-0.4
48	8C_ROI	-3.53	-23.33	-36.69	-9.97	0.001050636	0.0131	-0.4
49	STV_ROI	-3.99	-14.88	-22.41	-7.36	0.000263207	0.0119	-0.4
50	A4_ROI	-4.06	-17.88	-26.77	-8.99	0.00021393	0.0114	-0.4
51	TE1p_ROI	-5.71	-14.83	-20.08	-9.59	1.10339E-06	0.0056	-0.4
52	MBelt_ROI	-5.08	-12.79	-17.86	-7.71	8.55371E-06	0.0075	-0.4
53	10v_ROI	-4.65	-8.69	-12.46	-4.92	3.42731E-05	0.0081	-0.4
54	9m_ROI	-5.89	-17.48	-23.47	-11.49	6.14481E-07	0.0050	-0.4
55	OP1_ROI	-4.05	-10.86	-16.27	-5.44	0.000221589	0.0117	-0.5
56	TE1a_ROI	-4.54	-11.90	-17.20	-6.61	4.88419E-05	0.0083	-0.5
57	a10p_ROI	-4.25	-5.40	-7.97	-2.84	0.000120838	0.0106	-0.5
58	TF_ROI	-4.46	-17.81	-25.87	-9.75	6.19501E-05	0.0086	-0.5
59	9a_ROI	-5.63	-11.00	-14.95	-7.05	1.46897E-06	0.0058	-0.5
60	IFJp_ROI	-4.35	-10.67	-15.62	-5.71	8.83672E-05	0.0097	-0.5
61	a47r_ROI	-4.35	-12.48	-18.27	-6.69	8.77598E-05	0.0094	-0.5
62	IFSp_ROI	-5.41	-17.19	-23.61	-10.77	3.00613E-06	0.0067	-0.5
63	IFJa_ROI	-5.13	-17.74	-24.72	-10.76	7.29695E-06	0.0072	-0.5
64	STSdp_ROI	-5.95	-35.19	-47.13	-23.25	5.09321E-07	0.0044	-0.5
65	PGs_ROI	-5.91	-16.05	-21.53	-10.57	5.75502E-07	0.0047	-0.5
66	PGi_ROI	-6.95	-70.83	-91.42	-50.25	1.93743E-08	0.0031	-0.6
67	8Av_ROI	-5.76	-22.17	-29.94	-14.39	9.49161E-07	0.0053	-0.6
68	47l_ROI	-6.25	-20.88	-27.63	-14.13	1.90844E-07	0.0036	-0.6
69	PHT_ROI	-5.46	-13.07	-17.91	-8.23	2.55835E-06	0.0064	-0.6
70	OP2_3_ROI	-5.60	-11.07	-15.06	-7.08	1.60231E-06	0.0061	-0.6
71	p47r_ROI	-5.14	-12.45	-17.35	-7.56	7.14522E-06	0.0069	-0.6
72	STGa_ROI	-7.82	-12.38	-15.58	-9.19	1.16034E-09	0.0022	-0.6
73	31pd_ROI	-6.23	-13.02	-17.25	-8.80	2.06755E-07	0.0039	-0.6
74	IFSa_ROI	-6.08	-13.43	-17.89	-8.97	3.28848E-07	0.0042	-0.7
75	44_ROI	-7.49	-30.12	-38.24	-22.00	3.35745E-09	0.0025	-0.7
76	STSvp_ROI	-7.25	-49.88	-63.77	-35.99	7.30193E-09	0.0028	-0.7
77	9p_ROI	-4.85	-12.95	-18.34	-7.56	1.7999E-05	0.0078	-0.8
78	55b_ROI	-8.56	-37.93	-46.87	-28.98	1.12761E-10	0.0019	-0.8
79	PSL_ROI	-8.85	-58.81	-72.22	-45.40	4.60536E-11	0.0017	-0.8
80	SFL_ROI	-9.74	-42.57	-51.40	-33.74	3.1677E-12	0.0014	-0.8
81	45_ROI	-11.26	-56.69	-66.86	-46.52	4.02E-14	0.0006	-0.9
82	PBelt_ROI	-12.03	-51.76	-60.45	-43.07	4.9E-15	0.0003	-0.9
83	STSva_ROI	-9.80	-37.12	-44.77	-29.47	2.6732E-12	0.0011	-0.9

In the STORY-MATH contrast, 99 MMP regions showed significant differences (corrected p values) in the PE number between the hemispheres (Table 8, Figure 6B). Regions with an absolute ξ value equal to or greater than 0.5 are shown in Figure 6E. The area with significantly more PEs in the left hemisphere and expressing the largest absolute ξ value was the area STSv anterior (STSva_ROI, located within Brodmann 22), with a ξ = 1 and a mean difference value of 54.69 (CI 95% 49.34 ~ 60.04). The area with significantly more PEs in the right hemisphere and expressing the largest absolute ξ value was the Area STSd anterior (STSda_ROI, located within Brodmann 22), with a ξ = 0.8 and a mean difference value of 36.29 (CI 95% 28.16 ~ 44.41). For more information about the regions' location and name description, please refer to Figure 1.

**Table 8:** The difference in the positive element (PE) numbers in each MMP region between the two hemispheres in the STORY-MATH contrast in the right-handed group only. Data are presented in descending order of the explanatory measure of effect size (ξ). Results with positive effect size are in the direction of the right hemisphere, while negative results are in the direction of the left hemisphere. Only regions with a corrected p ≤ 0.05 are shown. The results were analyzed using the robust paired-sample t-test. MMP: Multimodal parcellation

	MMP Region	Statistics	Mean Difference	95% CI		p	p (corrected)	ξ
				Lower	Upper			
1	STSda_ROI	9.0169	36.29	28.16	44.41	2.79281E-11	0.0039	0.8
2	Ig_ROI	6.60	20.98	14.56	27.39	6.06466E-08	0.0058	0.7
3	PFcm_ROI	6.18	39.12	26.34	51.90	2.40023E-07	0.0072	0.6
4	6v_ROI	5.32	8.88	5.51	12.25	3.99039E-06	0.0106	0.5
5	VMV2_ROI	6.13	11.14	7.47	14.81	2.84198E-07	0.0078	0.5
6	LIPv_ROI	4.74	10.83	6.22	15.45	2.555E-05	0.0119	0.5
7	AIP_ROI	3.95	11.07	5.41	16.74	0.000303727	0.0158	0.4
8	OP4_ROI	4.59	22.90	12.82	32.99	4.17485E-05	0.0128	0.4
9	TGv_ROI	4.35	11.86	6.36	17.36	8.6854E-05	0.0142	0.4
10	7PC_ROI	4.39	14.83	8.01	21.66	7.75033E-05	0.0133	0.4
11	1_ROI	4.94	34.64	20.48	48.80	1.35811E-05	0.0114	0.4
12	OFC_ROI	4.11	18.40	9.37	27.44	0.000182524	0.0153	0.4
13	24dd_ROI	3.50	14.60	6.17	23.02	0.001147018	0.0183	0.4
14	2_ROI	4.13	42.05	21.47	62.62	0.000175227	0.0150	0.4
15	6d_ROI	4.17	11.02	5.69	16.36	0.000151881	0.0147	0.4
16	FOP2_ROI	4.03	8.52	4.25	12.80	0.000237784	0.0156	0.4
17	A5_ROI	2.91	17.26	5.28	29.24	0.005815426	0.0222	0.3
18	v23ab_ROI	5.02	10.43	6.24	14.62	1.03837E-05	0.0111	0.3
19	RI_ROI	3.41	18.29	7.46	29.11	0.001464941	0.0192	0.3
20	p10p_ROI	2.72	6.10	1.58	10.62	0.009449721	0.0244	0.3
21	3a_ROI	4.77	22.21	12.81	31.62	2.33925E-05	0.0117	0.3
22	pOFC_ROI	2.42	5.26	0.87	9.65	0.019964854	0.0272	0.3
23	V3_ROI	4.37	20.21	10.88	29.55	8.24619E-05	0.0139	0.3
24	VIP_ROI	3.34	6.02	2.38	9.67	0.001816652	0.0194	0.3
25	TA2_ROI	2.73	6.21	1.61	10.81	0.009332141	0.0239	0.3
26	5m_ROI	3.42	11.31	4.64	17.98	0.001413199	0.0189	0.3
27	s6_8_ROI	2.44	2.74	0.47	5.00	0.019005655	0.0269	0.3
28	V4t_ROI	3.22	5.29	1.97	8.60	0.002481233	0.0200	0.3
29	4_ROI	3.94	46.83	22.80	70.87	0.000314442	0.0161	0.3
30	MST_ROI	3.15	8.00	2.87	13.13	0.003042541	0.0206	0.3
31	FOP1_ROI	3.20	3.60	1.33	5.87	0.002661502	0.0203	0.3
32	43_ROI	2.80	10.19	2.84	17.54	0.007768912	0.0231	0.3
33	PF_ROI	2.92	17.74	5.47	30.01	0.005664414	0.0219	0.3
34	6mp_ROI	2.90	13.71	4.18	23.25	0.005911687	0.0225	0.3
35	PFop_ROI	3.26	12.10	4.61	19.58	0.002219076	0.0197	0.3
36	p24_ROI	2.66	2.05	0.49	3.60	0.011229784	0.0253	0.3
37	VMV1_ROI	3.13	5.40	1.92	8.89	0.003224265	0.0208	0.3
38	FOP3_ROI	2.83	5.69	1.63	9.75	0.007128591	0.0228	0.3
39	SCEF_ROI	2.51	3.17	0.62	5.72	0.016255489	0.0261	0.2
40	LO3_ROI	3.12	6.88	2.43	11.33	0.003268718	0.0211	0.2
41	3b_ROI	3.06	26.79	9.11	44.46	0.003882296	0.0214	0.2
42	PFt_ROI	2.66	10.64	2.55	18.73	0.01120307	0.0250	0.2
43	V3CD_ROI	2.46	7.45	1.34	13.56	0.018028464	0.0267	0.2
44	LO1_ROI	2.72	3.93	1.02	6.84	0.009449252	0.0242	0.2
45	V6_ROI	2.48	5.26	0.97	9.55	0.017493734	0.0264	0.2
46	PCV_ROI	-2.54	-6.57	-11.80	-1.34	0.015115339	0.0258	-0.2
47	a24_ROI	-2.74	-3.98	-6.91	-1.04	0.009097161	0.0236	-0.2
48	MT_ROI	-2.33	-4.71	-8.81	-0.62	0.025102907	0.0275	-0.3
49	8C_ROI	-2.63	-15.05	-26.59	-3.50	0.011893717	0.0256	-0.3
50	52_ROI	-2.71	-4.26	-7.44	-1.09	0.009761512	0.0247	-0.3
51	POS2_ROI	-4.41	-7.76	-11.31	-4.21	7.21959E-05	0.0131	-0.3
52	a9_46v_ROI	-3.03	-1.90	-3.17	-0.64	0.004193235	0.0217	-0.3
53	POS1_ROI	-3.91	-12.98	-19.67	-6.28	0.000335991	0.0164	-0.3
54	H_ROI	-3.48	-11.76	-18.58	-4.94	0.001194375	0.0186	-0.3
55	10r_ROI	-3.88	-9.10	-13.83	-4.36	0.000375746	0.0167	-0.3
56	46_ROI	-4.35	-6.38	-9.34	-3.42	8.7553E-05	0.0144	-0.3
57	IP1_ROI	-2.77	-2.02	-3.50	-0.55	0.00829895	0.0233	-0.4
58	p24pr_ROI	-3.69	-7.31	-11.31	-3.31	0.00065503	0.0172	-0.4
59	TGd_ROI	-4.38	-36.14	-52.79	-19.49	7.92191E-05	0.0136	-0.4
60	31a_ROI	-3.65	-5.45	-8.47	-2.43	0.00074377	0.0178	-0.4
61	a24pr_ROI	-3.52	-5.93	-9.33	-2.53	0.001077439	0.0181	-0.4
62	31pv_ROI	-4.74	-17.50	-24.96	-10.04	2.61518E-05	0.0122	-0.4
63	a10p_ROI	-3.68	-8.71	-13.50	-3.93	0.000674146	0.0175	-0.4
64	10d_ROI	-5.38	-17.95	-24.70	-11.21	3.31724E-06	0.0103	-0.5
65	9a_ROI	-5.81	-21.57	-29.06	-14.08	7.95091E-07	0.0083	-0.5
66	10v_ROI	-8.64	-16.83	-20.77	-12.90	8.83966E-11	0.0042	-0.5
67	55b_ROI	-5.24	-16.29	-22.56	-10.01	5.08488E-06	0.0108	-0.5
68	A4_ROI	-5.39	-27.36	-37.61	-17.11	3.18104E-06	0.0100	-0.5
69	8BM_ROI	-5.43	-11.29	-15.48	-7.09	2.7915E-06	0.0094	-0.5
70	PBelt_ROI	-4.70	-19.31	-27.61	-11.01	2.95592E-05	0.0125	-0.5
71	TF_ROI	-5.86	-31.74	-42.68	-20.79	6.93286E-07	0.0081	-0.5
72	i6_8_ROI	-3.82	-3.43	-5.24	-1.62	0.000438112	0.0169	-0.5
73	p47r_ROI	-5.63	-9.90	-13.46	-6.35	1.44691E-06	0.0092	-0.5
74	44_ROI	-6.23	-17.14	-22.70	-11.59	2.00608E-07	0.0069	-0.5
75	PHA2_ROI	-5.39	-9.55	-13.13	-5.97	3.17609E-06	0.0097	-0.6
76	PHA3_ROI	-6.27	-15.33	-20.27	-10.39	1.80723E-07	0.0067	-0.6
77	STV_ROI	-5.74	-21.90	-29.61	-14.20	9.97612E-07	0.0089	-0.6
78	47l_ROI	-6.92	-23.90	-30.88	-16.93	2.11503E-08	0.0053	-0.6
79	STGa_ROI	-7.20	-10.69	-13.69	-7.69	8.58017E-09	0.0050	-0.6
80	d32_ROI	-5.77	-8.43	-11.38	-5.48	9.11489E-07	0.0086	-0.6
81	STSdp_ROI	-6.88	-45.88	-59.36	-32.40	2.46837E-08	0.0056	-0.6
82	SFL_ROI	-6.45	-24.31	-31.92	-16.70	9.94625E-08	0.0061	-0.6
83	PSL_ROI	-6.16	-25.86	-34.33	-17.38	2.55158E-07	0.0075	-0.6
84	TE1a_ROI	-10.30	-27.21	-32.55	-21.88	6.106E-13	0.0028	-0.7
85	TE1m_ROI	-6.27	-9.57	-12.65	-6.49	1.78897E-07	0.0064	-0.7
86	9m_ROI	-10.47	-45.31	-54.05	-36.57	3.748E-13	0.0019	-0.7
87	31pd_ROI	-8.43	-31.12	-38.57	-23.67	1.69624E-10	0.0044	-0.7
88	PHT_ROI	-7.42	-24.62	-31.32	-17.92	4.18414E-09	0.0047	-0.7
89	d23ab_ROI	-10.02	-19.88	-23.89	-15.88	1.3727E-12	0.0031	-0.8
90	PFm_ROI	-10.43	-41.52	-49.56	-33.48	4.194E-13	0.0022	-0.8
91	a47r_ROI	-9.43	-31.74	-38.53	-24.94	7.9412E-12	0.0036	-0.8
92	8Av_ROI	-10.01	-51.60	-62.01	-41.18	1.4433E-12	0.0033	-0.8
93	PGi_ROI	-12.61	-124.90	-144.91	-104.90	1.1E-15	0.0011	-0.8
94	TE1p_ROI	-10.39	-34.81	-41.58	-28.04	4.747E-13	0.0025	-0.8
95	9p_ROI	-10.95	-41.90	-49.63	-34.18	9.59E-14	0.0017	-0.9
96	45_ROI	-17.36	-60.43	-67.46	-53.40	1E-16	0.0006	-0.9
97	STSvp_ROI	-11.98	-72.83	-85.11	-60.56	5.6E-15	0.0014	-0.9
98	PGs_ROI	-14.02	-85.79	-98.14	-73.43	1E-16	0.0003	-0.9
99	STSva_ROI	-20.63	-54.69	-60.04	-49.34	1E-16	0.0008	-1.0

Left-Handed Group (n = 70)

In the STORY contrast, 67 MMP regions showed significant differences (corrected p values) in the PE number between the hemispheres (Table 9, Figure 5C). Regions with an absolute ξ value equal to or greater than 0.5 are shown in Figure 5F. The area with significantly more PEs in the left hemisphere and expressing the largest absolute ξ value was the Para Belt complex (PBelt_ROI, located within Brodmann 22 and 42), with a ξ = 0.9 and a mean difference value of 39.79 (CI 95% 32.95 ~ 46.62). The area with significantly more PEs in the right hemisphere and expressing the largest absolute ξ value was the A5 complex (A5_ROI, located within Brodmann 22), with a ξ = 0.9 and a mean difference value of 55.45 (CI 95% 48.17 ~ 62.74).

**Table 9:** The difference in the positive element (PE) numbers in each MMP region between the two hemispheres in the STORY contrast in the left-handed subjects only. Data are presented in descending order of the explanatory measure of effect size (ξ). Results with positive effect size are in the direction of the right hemisphere, while negative results are in the direction of the left hemisphere. Only regions with a corrected p ≤ 0.05 are shown. The results were analyzed using the robust paired-sample t-test. MMP: Multimodal parcellation

	MMP Region	Statistics	Mean Difference	95% CI		p	p (corrected)	ξ
				Lower	Upper			
1	A5_ROI	15.37	55.45	48.17	62.74	1E-16	0.0003	0.9
2	Ig_ROI	4.74	9.26	5.31	13.21	2.60941E-05	0.0039	0.6
3	pOFC_ROI	3.67	7.05	3.17	10.92	0.000684654	0.0081	0.5
4	p10p_ROI	5.76	6.55	4.25	8.84	9.58559E-07	0.0025	0.5
5	TPOJ1_ROI	3.82	33.81	15.95	51.67	0.000440628	0.0075	0.4
6	STSda_ROI	3.97	20.26	9.97	30.56	0.000279084	0.0067	0.4
7	TA2_ROI	3.89	10.55	5.08	16.02	0.000356854	0.0069	0.4
8	VMV2_ROI	4.16	4.67	2.40	6.93	0.000158122	0.0053	0.4
9	V4t_ROI	4.94	4.60	2.72	6.47	1.36625E-05	0.0033	0.4
10	5m_ROI	4.61	7.29	4.09	10.48	3.90026E-05	0.0042	0.3
11	V8_ROI	3.63	3.86	1.71	6.00	0.000783294	0.0083	0.3
12	FOP1_ROI	3.81	2.83	1.33	4.34	0.000458817	0.0078	0.3
13	25_ROI	2.59	3.38	0.75	6.01	0.013077303	0.0172	0.3
14	s6_8_ROI	3.14	2.57	0.92	4.22	0.003094052	0.0111	0.3
15	MST_ROI	3.47	4.71	1.97	7.46	0.001238465	0.0092	0.3
16	47s_ROI	3.47	7.83	3.27	12.39	0.001238603	0.0094	0.3
17	OFC_ROI	3.03	9.55	3.18	15.91	0.004223582	0.0117	0.3
18	PIT_ROI	2.99	3.95	1.28	6.62	0.004732791	0.0125	0.3
19	11l_ROI	2.49	4.17	0.79	7.55	0.0169465	0.0181	0.3
20	RSC_ROI	3.24	3.38	1.27	5.49	0.002397078	0.0103	0.3
21	24dv_ROI	2.79	6.10	1.68	10.51	0.008041266	0.0158	0.3
22	LO3_ROI	2.75	2.43	0.64	4.21	0.008835899	0.0164	0.3
23	V3_ROI	4.88	16.55	9.70	23.40	1.65827E-05	0.0036	0.3
24	5mv_ROI	2.92	6.29	1.94	10.63	0.005617968	0.0139	0.3
25	LO1_ROI	3.11	2.76	0.97	4.56	0.003394287	0.0114	0.3
26	7Pm_ROI	2.45	4.36	0.76	7.95	0.018718808	0.0189	0.2
27	v23ab_ROI	2.45	3.31	0.58	6.03	0.018510205	0.0186	0.2
28	PGp_ROI	2.57	4.26	0.91	7.61	0.013962041	0.0178	0.2
29	V3CD_ROI	2.92	4.21	1.30	7.13	0.005607613	0.0136	0.2
30	V2_ROI	2.66	12.67	3.05	22.29	0.011137293	0.0167	0.1
31	LIPd_ROI	-2.95	-3.21	-5.41	-1.02	0.005185957	0.0128	-0.2
32	SCEF_ROI	-2.94	-9.43	-15.90	-2.96	0.005331229	0.0131	-0.2
33	IFJp_ROI	-2.60	-3.98	-7.06	-0.89	0.012844825	0.0169	-0.2
34	TE1a_ROI	-2.81	-7.40	-12.73	-2.08	0.007628393	0.0153	-0.2
35	PF_ROI	-2.57	-10.38	-18.53	-2.23	0.013820501	0.0175	-0.2
36	PGi_ROI	-2.75	-35.81	-62.11	-9.51	0.008823351	0.0161	-0.3
37	a9_46v_ROI	-2.85	-2.05	-3.50	-0.60	0.006761752	0.0147	-0.3
38	a47r_ROI	-3.02	-8.21	-13.71	-2.72	0.004372908	0.0122	-0.3
39	d23ab_ROI	-3.02	-2.43	-4.05	-0.81	0.004302289	0.0119	-0.3
40	d32_ROI	-2.91	-3.43	-5.81	-1.05	0.005825856	0.0142	-0.3
41	MBelt_ROI	-2.84	-7.90	-13.53	-2.28	0.007036751	0.0150	-0.3
42	31pd_ROI	-2.93	-7.14	-12.07	-2.21	0.005578968	0.0133	-0.3
43	9_46d_ROI	-3.62	-7.17	-11.17	-3.17	0.000805537	0.0086	-0.3
44	PGs_ROI	-2.91	-11.14	-18.88	-3.41	0.005826056	0.0144	-0.3
45	OP1_ROI	-2.80	-7.74	-13.31	-2.16	0.007705141	0.0156	-0.3
46	A1_ROI	-3.25	-6.74	-10.93	-2.55	0.002310036	0.0100	-0.4
47	8Av_ROI	-3.19	-13.48	-22.00	-4.95	0.00270986	0.0106	-0.4
48	OP2_3_ROI	-3.16	-7.17	-11.74	-2.59	0.002949562	0.0108	-0.4
49	10v_ROI	-4.16	-6.05	-8.98	-3.11	0.000158777	0.0056	-0.4
50	FOP4_ROI	-3.29	-8.40	-13.57	-3.24	0.002090477	0.0097	-0.4
51	STV_ROI	-3.54	-13.48	-21.17	-5.78	0.001017831	0.0089	-0.4
52	9m_ROI	-4.57	-18.45	-26.60	-10.30	4.38438E-05	0.0044	-0.4
53	A4_ROI	-4.03	-19.24	-28.87	-9.60	0.0002348	0.0061	-0.4
54	9a_ROI	-3.83	-10.19	-15.56	-4.82	0.000426179	0.0072	-0.4
55	p47r_ROI	-4.28	-8.14	-11.99	-4.30	0.000110678	0.0047	-0.4
56	PHT_ROI	-3.99	-12.45	-18.75	-6.16	0.00026267	0.0064	-0.4
57	9p_ROI	-4.27	-8.05	-11.85	-4.24	0.000112864	0.0050	-0.5
58	47l_ROI	-5.26	-17.71	-24.52	-10.91	4.86094E-06	0.0031	-0.5
59	44_ROI	-4.11	-25.52	-38.07	-12.98	0.000185038	0.0058	-0.5
60	STGa_ROI	-5.35	-10.79	-14.85	-6.72	3.57536E-06	0.0028	-0.6
61	55b_ROI	-6.73	-33.60	-43.67	-23.52	3.92826E-08	0.0019	-0.7
62	SFL_ROI	-6.44	-33.60	-44.13	-23.06	1.01429E-07	0.0022	-0.7
63	STSvp_ROI	-7.38	-42.07	-53.58	-30.56	4.82296E-09	0.0017	-0.7
64	PSL_ROI	-7.44	-47.48	-60.36	-34.59	3.98101E-09	0.0014	-0.7
65	45_ROI	-7.45	-44.36	-56.39	-32.33	3.88896E-09	0.0011	-0.8
66	STSva_ROI	-9.30	-35.60	-43.32	-27.87	1.17764E-11	0.0008	-0.9
67	PBelt_ROI	-11.76	-39.79	-46.62	-32.95	1.02E-14	0.0006	-0.9

In the STORY-MATH contrast, 76 MMP regions showed significant differences (corrected p values) in the PE number between both hemispheres (Table 10, Figure 6C). Regions with an absolute ξ value equal to or greater than 0.5 are shown in Figure 6F. The area with significantly more PEs in the left hemisphere and expressing the largest absolute ξ value was the Area STSv anterior (STSva_ROI, located within Brodmann 22), with a ξ = 1 and a mean difference value of 58.98 (CI 95% 54.04 ~ 63.91). The area with significantly more PEs in the right hemisphere and expressing the largest absolute ξ value was the area STSd anterior (STSda_ROI, located within Brodmann 22), with a ξ = 1 and a mean difference value of 45.50 (CI 95% 38.13 ~ 52.87). For more information about the regions' location and name description, please refer to Figure 1.

**Table 10:** The difference in the positive element (PE) numbers in each MMP region between the two hemispheres in the STORY-MATH contrast in the left-handed group only. Data are presented in descending order of the explanatory measure of effect size (ξ). Results with positive effect size are in the direction of the right hemisphere, while negative results are in the direction of the left hemisphere. Only regions with a corrected p ≤ 0.05 are shown. The results were analyzed using the robust paired-sample t-test. MMP: Multimodal parcellation

	MMP Region	Statistics	Mean Difference	95% CI		p	p (corrected)	ξ
				Lower	Upper			
1	STSda_ROI	12.46	45.50	38.13	52.87	1.6E-15	0.0006	1.0
2	Ig_ROI	5.63	18.33	11.75	24.92	1.4739E-06	0.0042	0.7
3	s6_8_ROI	3.99	5.31	2.62	8.00	0.000268841	0.0100	0.6
4	TPOJ1_ROI	4.22	45.79	23.88	67.69	0.000131339	0.0089	0.6
5	1_ROI	7.09	45.95	32.87	59.04	1.22454E-08	0.0025	0.5
6	p10p_ROI	4.39	10.98	5.93	16.02	7.7356E-05	0.0078	0.5
7	A5_ROI	4.27	21.62	11.39	31.84	0.000112848	0.0083	0.5
8	AIP_ROI	3.28	6.12	2.36	9.88	0.002097538	0.0144	0.4
9	VMV2_ROI	3.59	8.60	3.76	13.43	0.000870916	0.0117	0.4
10	TE2p_ROI	3.89	13.21	6.35	20.08	0.000361871	0.0103	0.4
11	pOFC_ROI	4.12	6.76	3.45	10.07	0.000177376	0.0094	0.4
12	PFcm_ROI	4.70	21.52	12.28	30.77	2.89971E-05	0.0061	0.4
13	PF_ROI	4.09	25.00	12.66	37.34	0.000195969	0.0097	0.4
14	TGv_ROI	3.19	10.19	3.73	16.65	0.002763319	0.0156	0.4
15	6v_ROI	3.44	5.55	2.30	8.80	0.001331088	0.0128	0.4
16	5m_ROI	4.69	11.98	6.82	17.13	2.97921E-05	0.0064	0.4
17	2_ROI	5.18	41.12	25.08	57.16	6.32736E-06	0.0050	0.4
18	LIPv_ROI	4.25	8.69	4.56	12.82	0.000120428	0.0086	0.4
19	OP4_ROI	3.43	18.79	7.71	29.86	0.001405505	0.0131	0.3
20	V3CD_ROI	4.45	9.79	5.34	14.23	6.53585E-05	0.0075	0.3
21	FOP5_ROI	3.30	5.67	2.20	9.13	0.001998338	0.0142	0.3
22	V3_ROI	5.71	23.88	15.43	32.33	1.1181E-06	0.0039	0.3
23	OFC_ROI	3.38	15.24	6.15	24.33	0.001581677	0.0133	0.3
24	8BL_ROI	2.88	14.52	4.33	24.71	0.006320197	0.0181	0.3
25	LO1_ROI	3.10	5.52	1.93	9.12	0.003491747	0.0158	0.3
26	v23ab_ROI	3.54	9.12	3.92	14.32	0.001001612	0.0122	0.3
27	LO3_ROI	3.19	6.81	2.51	11.11	0.002691118	0.0153	0.3
28	43_ROI	3.35	10.26	4.08	16.45	0.00173546	0.0136	0.3
29	VMV1_ROI	2.76	4.71	1.27	8.16	0.008592925	0.0197	0.3
30	PoI2_ROI	2.75	9.52	2.52	16.53	0.008933074	0.0200	0.3
31	24dd_ROI	3.06	11.36	3.85	18.86	0.003946076	0.0161	0.3
32	6d_ROI	2.82	7.05	1.99	12.10	0.007459265	0.0183	0.3
33	MST_ROI	2.76	7.62	2.05	13.19	0.008518853	0.0194	0.3
34	TA2_ROI	2.78	6.31	1.73	10.89	0.008089743	0.0189	0.3
35	4_ROI	3.20	27.33	10.10	44.57	0.002636226	0.0147	0.2
36	PGp_ROI	2.79	7.95	2.19	13.71	0.007974927	0.0186	0.2
37	3a_ROI	2.89	13.07	3.93	22.21	0.006178176	0.0178	0.2
38	V2_ROI	2.95	13.14	4.14	22.14	0.005241938	0.0169	0.2
39	a24_ROI	-2.68	-4.36	-7.64	-1.08	0.01045318	0.0203	-0.2
40	31a_ROI	-2.41	-3.76	-6.91	-0.62	0.020309931	0.0211	-0.3
41	TGd_ROI	-2.94	-24.45	-41.24	-7.66	0.005355108	0.0172	-0.3
42	PHA2_ROI	-2.50	-5.40	-9.77	-1.04	0.016541084	0.0208	-0.3
43	FOP4_ROI	-2.61	-1.95	-3.46	-0.44	0.012686076	0.0206	-0.3
44	10v_ROI	-2.89	-7.74	-13.14	-2.33	0.006124376	0.0175	-0.3
45	52_ROI	-3.04	-4.95	-8.25	-1.66	0.004161147	0.0167	-0.3
46	10d_ROI	-3.49	-12.43	-19.62	-5.23	0.001174402	0.0125	-0.3
47	STV_ROI	-3.20	-14.38	-23.46	-5.30	0.00266218	0.0150	-0.3
48	55b_ROI	-3.05	-11.26	-18.73	-3.80	0.004040863	0.0164	-0.3
49	PHA3_ROI	-3.35	-8.62	-13.82	-3.42	0.001748816	0.0139	-0.3
50	STSdp_ROI	-2.78	-23.38	-40.39	-6.37	0.00826259	0.0192	-0.3
51	PBelt_ROI	-4.62	-16.50	-23.71	-9.29	3.75863E-05	0.0069	-0.4
52	47l_ROI	-3.56	-13.02	-20.41	-5.64	0.00095113	0.0119	-0.4
53	A4_ROI	-4.71	-23.14	-33.06	-13.22	2.82596E-05	0.0058	-0.4
54	9a_ROI	-5.26	-23.14	-32.03	-14.26	4.82596E-06	0.0044	-0.5
55	TE1m_ROI	-3.61	-5.71	-8.91	-2.52	0.000829179	0.0114	-0.5
56	a47r_ROI	-4.64	-19.40	-27.85	-10.96	3.52863E-05	0.0067	-0.5
57	PSL_ROI	-3.84	-17.88	-27.29	-8.47	0.000420589	0.0106	-0.5
58	8BM_ROI	-4.21	-9.60	-14.19	-5.00	0.000134576	0.0092	-0.5
59	SFL_ROI	-3.61	-15.17	-23.65	-6.68	0.000823525	0.0111	-0.5
60	d32_ROI	-4.28	-7.60	-11.18	-4.01	0.000109289	0.0081	-0.5
61	PHT_ROI	-3.65	-14.26	-22.15	-6.38	0.000728756	0.0108	-0.5
62	TF_ROI	-4.84	-28.55	-40.46	-16.63	1.88349E-05	0.0056	-0.5
63	9m_ROI	-8.52	-42.60	-52.69	-32.50	1.29057E-10	0.0019	-0.6
64	TE1p_ROI	-4.59	-17.95	-25.84	-10.06	4.09198E-05	0.0072	-0.6
65	PFm_ROI	-5.05	-34.14	-47.78	-20.50	9.40088E-06	0.0053	-0.6
66	STGa_ROI	-6.25	-9.43	-12.47	-6.38	1.90183E-07	0.0036	-0.6
67	31pd_ROI	-6.31	-28.07	-37.05	-19.09	1.54402E-07	0.0033	-0.6
68	8Av_ROI	-5.24	-35.95	-49.81	-22.10	5.1592E-06	0.0047	-0.6
69	TE1a_ROI	-6.74	-26.57	-34.54	-18.61	3.86712E-08	0.0028	-0.7
70	d23ab_ROI	-9.64	-15.55	-18.80	-12.29	4.2366E-12	0.0011	-0.7
71	9p_ROI	-7.87	-35.95	-45.18	-26.72	1.01295E-09	0.0022	-0.7
72	PGi_ROI	-6.52	-87.31	-114.34	-60.28	7.78713E-08	0.0031	-0.8
73	STSvp_ROI	-9.03	-58.00	-70.98	-45.02	2.71769E-11	0.0017	-0.9
74	45_ROI	-9.42	-47.19	-57.31	-37.08	8.1817E-12	0.0014	-0.9
75	PGs_ROI	-11.93	-68.31	-79.88	-56.74	6.7E-15	0.0008	-0.9
76	STSva_ROI	-24.12	-58.98	-63.91	-54.04	1E-16	0.0003	-1.0

Differences between the handedness groups

In the Entire Brain Surface

**Figure 7:**
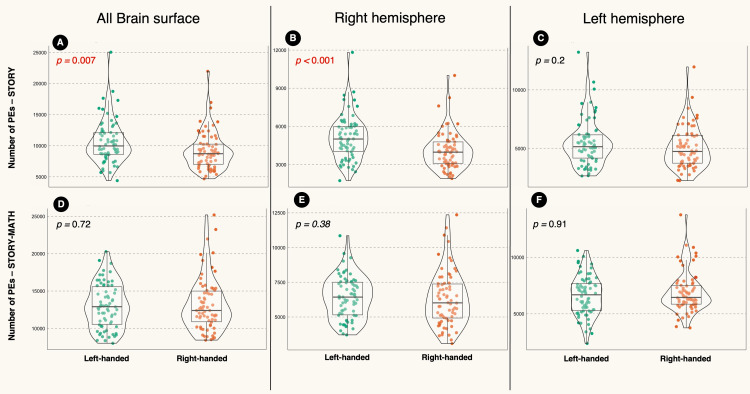
Violin plot showing the difference between the two handedness groups. In the Story contrast, the LH group displayed significantly more PEs than the RH in the whole brain surface (A) and the right hemisphere (B), while the left hemisphere did not show significant differences between the two groups (C). The STORY-MATH did not show any difference between the two groups in the whole brain surface (D), the right hemisphere (E), and the left hemisphere (F). The results represent the outcomes of robust independent-sample t-test analyses. PEs: Positive elements; LH: left-handed; RH: right-handed

The STORY contrast showed a significant difference in the total number of PEs between the handedness groups (p = 0.007, Figure 7A), with the LH group having more PEs than the RH. In contrast, the STORY-MATH contrast showed no significant difference between the handedness groups (p = 0.72, Figure 7D).

In Each Hemisphere

The STORY contrast has shown only a significant difference in the number of PEs between the handedness groups in the right hemisphere (p < 0.001, Figure 7B), with the LH group having more PEs than the RH. However, no differences between the LH and RH groups were seen in the left hemisphere (p = 0.2, Figure 7C). The STORY-MATH contrast did not show any significant difference between the handedness groups in the right (p = 0.38, Figure 7E) or in the left hemisphere (p = 0.91, Figure 7F).

In the MMP Regions

**Figure 8:**
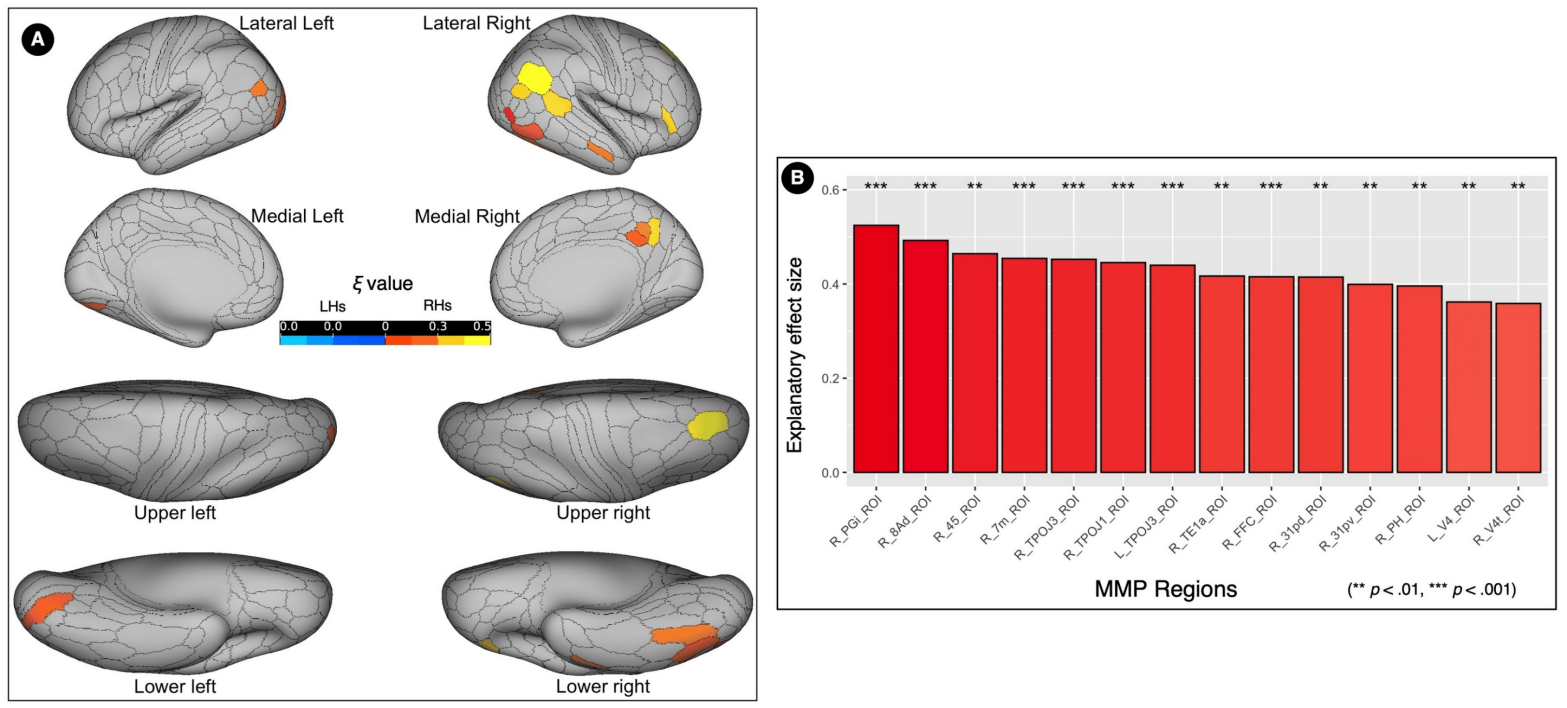
The explanatory measure of effect size (ξ) represents the mean difference in the number of positive elements (PEs) between handedness groups across 360 MMP regions in both hemispheres, using the STORY contrast data The 14 regions showing significant values are displayed on an inflated brain surface for both hemispheres (A) and in a bar chart (B). All 14 regions exhibited significantly higher values in the left-handed (LH) group only, with 12 regions located in the right hemisphere. The ξ and p-value are measured using robust independent-sample t-test analyses.

In the STORY contrast, 14 MMP regions showed a significant difference (corrected p-value) between the handedness groups (Table 11). All 14 regions showed significantly more PEs, but only in the LH group. Furthermore, 12 out of the 14 regions are on the right hemisphere (Figure 8). The area with significantly more PEs and expressing the largest absolute ξ value was the right Area PGi (R_PGi_ROI, located within Brodmann 39 and 40), with a ξ = 0.52 and a mean difference value of 64.45 (CI 95% 36.01 ~ 92.89). For more information about the regions' location and name description, please refer to Figure 1.

**Table 11:** The difference in the positive element (PE) numbers in each MMP region between the handedness groups in the STORY contrast. Results are in the descending order of the explanatory measure of effect size (ξ). Results with positive effect size are in the direction of left-handedness. Only regions with a corrected p ≤ 0.05 are shown. The results were analyzed using the robust independent-sample t-test. MMP: Multimodal parcellation

	MMP Region	Statistic	Mean Difference	95%CI		p	p (corrected)	ξ
				Lower	Upper			
1	R_PGi_ROI	4.51	64.45	36.01	92.89	0.00002	0.0001	0.52
2	R_8Ad_ROI	3.66	10.98	4.97	16.99	0.00056	0.0008	0.49
3	R_45_ROI	3.30	16.17	6.38	25.95	0.00156	0.0019	0.46
4	R_7m_ROI	4.50	16.81	9.35	24.27	0.00003	0.0003	0.45
5	R_TPOJ3_ROI	3.58	8.17	3.63	12.71	0.0006	0.0010	0.45
6	R_TPOJ1_ROI	3.96	39.83	19.80	59.87	0.00017	0.0006	0.45
7	L_TPOJ3_ROI	4.14	8.38	4.35	12.41	0.00009	0.0004	0.44
8	R_TE1a_ROI	3.30	9.10	3.60	14.59	0.00153	0.0018	0.42
9	R_FFC_ROI	3.73	11.00	5.13	16.87	0.00036	0.0007	0.42
10	R_31pd_ROI	3.52	6.10	2.63	9.56	0.00084	0.0014	0.41
11	R_31pv_ROI	3.54	4.31	1.89	6.73	0.00068	0.0011	0.40
12	R_PH_ROI	3.50	11.74	5.06	18.42	0.00079	0.0013	0.40
13	L_V4_ROI	3.30	22.52	8.94	36.11	0.00146	0.0015	0.36
14	R_V4t_ROI	3.29	4.00	1.58	6.42	0.0015	0.0017	0.36

The STORY-MATH contrast did not show any region with a difference between the two handedness groups.

## Discussion

This study explored language asymmetry variations among handedness groups, employing two distinct fMRI language task contrasts: STORY and STORY-MATH. The former delineates speech listening/comprehension activity, while the latter can target semantic processes [15]. Our examination of language dominance extended across both hemispheric and finer spatial scales, encompassing 180 regions (Figure 1). 

This study yielded two significant observations. First, irrespective of handedness type, conventionally recognized language regions within the brain-such as the inferior frontal and posterior perisylvian regions-exhibited notably more activity in the left hemisphere. Second, distinct differences surfaced when directly contrasting the handedness groups within the STORY contrast (speech comprehension). LH individuals showed significantly more cortical activity in specific areas within the right hemisphere, notably in the angular gyrus (AG) of the inferior parietal lobule (Brodmann 39 and 40).

Differences at the hemispheric level

We detected a significant discrepancy in the count of PEs between the left and right hemispheres across the entire sample, evident in both the STORY (p < 0.0001, Figure 4A) and STORY-MATH (p < 0.001, Figure 4D) contrasts, favoring higher PEs in the left hemisphere. A similar trend was noted within the RH subgroup, demonstrating a notably greater number of PEs in the left hemisphere for both STORY (p < 0.0001, Figure 4B) and STORY-MATH (p < 0.0001, Figure 4E) contrasts. These findings align with other previous studies. Vassal et al. identified global left lateralization of BOLD clusters during sentence comprehension tasks in 20 healthy RH subjects [24]. Likewise, a study on 30 healthy RH individuals during a semantic decision task reported predominantly left-sided brain activation, although bilateral activation of the superior temporal sulcus (STS) was observed [25]. Another investigation highlighted left inferior and middle frontal regions, temporal, and occipito-parietal areas as predominantly involved during an fMRI semantic task [26].

The whole sample and RH subgroup findings align with established language models. Peelle's hierarchical language asymmetry model anticipates left lateralization in connected language processing, emphasizing its reliance on intricate linguistic operations [27]. Price's anatomical model, derived from a comprehensive analysis of PET and fMRI studies spanning 20 years, identifies multiple left hemisphere areas engaged in various elements and tasks associated with speech comprehension and semantics, albeit without precise anatomical localization [28]. Poeppel proposed an alternative hypothesis, indicating left hemisphere dominance in complex linguistic operations, while bilateral processing is pivotal for auditory perception and speech production. On the other hand, the right hemisphere is implicated in non-linguistic aspects of auditory perception and musical processing [29]. These models collectively bolster our observed lateralization patterns, corroborating the dominance of the left hemisphere in language-related complexities observed within our study.

However, outcomes in the LH group subjects exhibited notable differences. No significant difference surfaced in the count of PEs between hemispheres in either the STORY contrast (p = 0.25, Figure 4C) or STORY-MATH (p = 0.28, Figure 4F) contrast, suggesting an absence of distinct hemispheric dominance. Similarly, an investigation assessing language laterality index (LI) reported a lack of clear dominance. Knecht et al. utilized functional TDS to assess language lateralization in 326 healthy individuals, revealing that 54% of LH persons (EHI < -75) exhibited right-lateralized language [6]. Their study noted a linear increase in right hemisphere language dominance with the degree of LH people’s EHI. However, Powell et al. examined the correlation between language laterality and EHI degree in 40 LH individuals and found no significant correlation [7]. These findings highlight the complexity of language dominance patterns among left-handed individuals, showing diverse outcomes in the relationship between handedness and language lateralization.

Additional studies have also evidenced left-lateralization in the language functions of most LH subjects. Szaflarski et al., in their study involving 50 healthy LH persons, observed cortical activation patterns during fMRI language listening to speech. They noted right hemispheric activation in 8%, symmetric activation in 14%, and predominant left hemispheric activation in 78% [30]. Likewise, Mazoyer et al. analyzed the hemispheric functional lateralization index (HFLI) in both handedness groups, noting a distinct left hemisphere dominance with positive HFLI values observed in 78% of LH [31].

The disparities in outcomes between our study and prior studies do not signify disagreements due to the differing approaches in analyzing laterality. In our investigation, we refrained from employing a cutoff value to classify language laterality, a standard practice in laterality index (LI) studies. LI methodology holds merits for its prevalence, simplicity, and facilitating inter-study comparisons using consistent methodologies and cutoffs. However, the arbitrary nature of these cutoffs across studies introduces variability. Moreover, LI can be influenced by sample size discrepancies and hemispheric variations, impeding standardized comparisons of typical and atypical lateralization proportions in different studies [32]. Our study, structured to scrutinize mean differences between target variables (e.g., RH and LH groups), opted for effect size and p-value over arbitrary cutoff thresholds. Utilizing effect size enabled us to quantify the magnitude of lateralization, veering away from presumptions of categorical phenomena common in LI analyses. Effect size, being comparable across diverse methodologies and sample sizes, provided a more nuanced approach to evaluate and compare lateralization strength across studies.

Differences in MMP regions in each contrast between the two hemispheres

Whole Sample (n =140)

We probed deeper into language laterality, analyzing 180 regions using the MMP atlas [12]. Analysis across the entire sample unveiled noteworthy differences in PE numbers between hemispheres across 103 regions in the STORY contrast (Table 5). Among these, 62% exhibited notably higher PE numbers in the left hemisphere, mirroring a leftward dominance akin to the broader hemisphere-level findings. Additionally, our assessment of effect size measures across MMP regions highlighted significantly elevated PEs in the commonly recognized language areas of the human brain's left hemisphere in contrast to the right (Figure 5A). Prominent MMP regions demonstrating significantly higher PEs on the left side encompassed the auditory association cortex, STS, inferior frontal gyrus (e.g., area 45), area 55b (Brodmann 6), superior frontal language area (SFL, Brodmann 6 and 8), and perisylvian language area (PSL, Brodmann 40 and 42) (Figure 5D). Notably, the SFL and PSL regions, newly identified language areas in the MMP publication, exhibit functional connections with various other language regions, such as areas 44/45 of the inferior frontal cortex, multiple regions within the superior temporal cortex, and area 55b [12]. Area 55b, also known as the Hopf area, holds significance as a critical integration hub for both dorsal and ventral language streams [33].

In the STORY-MATH contrast, notable differences in PE numbers between hemispheres emerged across 116 regions (Table 6). Of these, 47% exhibited significantly higher PEs in the left hemisphere, indicating a comparatively lesser dominance in semantic processing compared to speech comprehension. While the STORY-MATH contrast revealed fewer MMP regions favoring the left hemisphere in PE numbers than the STORY contrast, most recognized language areas maintained left-sided asymmetry (Figure 6A). Prominent MMP regions displaying elevated PEs in the left hemisphere included the STS, AG, and inferior frontal regions. However, regions within the supramarginal gyrus (SMG), specifically part of the inferior parietal lobule corresponding to Brodmann's area 40, showed significantly higher PEs in the right hemisphere compared to the left (Figure 6D). This deviation in lateralization highlights an intriguing shift within the SMG region during the semantic processing contrast.

RH Group (n =70)

In the exclusive analysis of the RH group, disparities in PE numbers between hemispheres were observed across 83 regions in the STORY contrast (Table 7). Notably, 75% of these regions exhibited significantly elevated PEs in the left hemisphere, revealing a heightened left dominance compared to the comprehensive sample. Consistently, well-recognized language areas displayed notably increased PEs in the left hemisphere (Figure 5B), mirroring patterns observed in the broader analysis (Figure 5D).

Transitioning to the STORY-MATH contrast in RH, notable distinctions in PE numbers emerged across 99 regions (Table 8). Among these regions, 55% displayed more PEs in the left hemisphere, indicating a subdued left dominance in semantic processing compared to the STORY contrast within the same subgroup. Correspondingly, predominant asymmetry towards the left was evident in most recognized language areas (Figure 6B). Leading MMP regions mirrored the patterns observed in the comprehensive sample analysis, exhibiting a higher number of PEs on the left or right sides.

LH Group (n =70)

In the LH group analysis, noticeable disparities in PE numbers between hemispheres were evident across 67 regions in the STORY contrast (Table 9). Approximately 55% of these regions displayed significantly elevated PEs in the left hemisphere, signifying the weakest left dominance within the STORY contrast (Figure 5C). However, recognized language regions continued to exhibit notably increased PEs in the left hemisphere compared to the right. The primary MMP regions displaying heightened PEs on the left mirrored those identified in the comprehensive sample and right-handed subgroup analyses (Figure 5F).

Shifting to the STORY-MATH contrast, noteworthy distinctions in PE numbers between both hemispheres emerged across 76 regions (Table 10). Of these regions, 50% showed notably higher PEs in the left hemisphere, implying a lack of hemispheric dominance in semantic processing compared to speech comprehension within this subgroup, consistent with earlier hemispheric analyses. Despite this, asymmetry toward the left persisted in differences within recognized language areas (Figure 6C). Leading MMP regions showcasing higher PEs on the left or right corresponded to those observed in the comprehensive sample analysis (Figure 6F).

Differences in each contrast between the handedness groups

In comparing the LH and RH groups, the STORY-MATH contrast displayed no notable differences across the entire brain surface or within each hemisphere (Figures 7D-7F). However, in the STORY contrast, the LH group exhibited significantly higher PEs compared to the RH (p = 0.007, Figure 7A). Further examination per hemisphere revealed a significant disparity, indicating the right hemisphere of LH had notably more PEs than the RHs (p < 0.001, Figure 7B). Conversely, no significant variance emerged between the handedness groups in the left hemisphere (p = 0.2, Figure 7C). The locations of these PEs within the right hemisphere warrant a closer MMP-level investigation.

At the MMP level, p-value correcting for multiple comparisons (360 tests) unveiled no significant differences in any region within the STORY-MATH contrast between the two handedness groups. However, in the STORY contrast, 14 MMP regions demonstrated a statistically significant difference (Table 11, Figure 8). Surprisingly, 12 out of the 14 regions were located in the right hemisphere. Interestingly, all 14 regions had significantly higher PEs only in the LH group, with none favoring the RHs. The identified regions within the right hemisphere predominantly encompassed the AG, dorsolateral prefrontal cortex (DLPFC), medial sub-parietal/posterior cingulate cortex, and inferior frontal regions (Figure 8). The region with the highest number of PEs was R_PGi_ROI, located in the right AG (Brodmann 39 and 40) (p = 0.0001, ξ = 0.52).

Is there asymmetrical language function dominance in LHs?

The investigation into asymmetrical language function dominance in the LH group revealed no specific hemispheric dominance. However, when examining differences at a lower spatial scale (MMP regions), the well-known language areas consistently retained their typical left-sided asymmetry (Figures 5C, 5F, 6C, 6F). Upon detailed region-to-region comparison between LH and RH during speech comprehension task (STORY contrast), several regions displayed notably heightened activity in LH individuals, primarily in the right hemisphere (Figure 8). Notably active areas included the AG, DLPFC, area 45, and sub-parietal/posterior cingulate cortex.

This study has limitations. One potential limitation lies in our selection bias within the RH sample. Our study focused solely on true RHs (EHI = 100), and we could not include an adequate number of true LHs (EHI = -100) from the HCP database. The 70 LHs analyzed had EHIs at or below -40, raising uncertainty about the comparability of true RHs with these LHs and its potential impact on the results. Moreover, the RH group had significantly more females. Future research involving RHs with varying EHI values could offer insights into how EHI influences language dominance at the MMP region level. Furthermore, exploring language dominance in ambidextrous individuals may present intriguing avenues for investigation.

Another limitation relates to our focus solely on the number of vertices exhibiting positive BOLD signals above a set threshold without considering signal intensity disparities between hemispheres. However, we opted for the mean z-statistics value as a threshold, aiming to directly reflect the impact of intensity changes in each region on the analysis outcome. This choice aimed to mitigate biases introduced by arbitrary thresholding and clustering methodologies.

That being said, the study has strengths. The strength lies in its comprehensive approach to exploring language asymmetry across handedness groups using advanced methodologies. By analyzing fMRI data with two distinct task contrasts (STORY and STORY-MATH), the research assessed language dominance at both hemispheric and fine-grained spatial levels, leveraging the Human Connectome Project's MMP atlas. It identified key differences in cortical activity between LH and RH individuals, revealing novel findings about lateralization patterns, particularly in left-handed individuals. Additionally, the use of effect size instead of traditional cutoff thresholds provided a nuanced evaluation of lateralization, ensuring robust and scalable comparisons across methodologies and sample sizes.

## Conclusions

In terms of speech comprehension, the conventional language regions in the left hemisphere retain dominance in both LH and RH individuals. Nevertheless, LH individuals exhibit heightened activity in specific areas of the right hemisphere compared to their RH counterparts, including the AG, DLPFC, medial sub-parietal/posterior cingulate cortex, and inferior frontal regions. Importantly, these distinctions were discernible only through scrutiny of smaller, more specific brain regions rather than conventional hemispheric analysis methods.

Future research should include a more diverse sample of LH and RH individuals, including those with varying EHI scores and ambidextrous participants, to explore the full spectrum of handedness and its impact on language lateralization. Studies could also investigate signal intensity differences between hemispheres, rather than focusing solely on active regions, for a deeper understanding of asymmetry. Additionally, exploring language dominance in larger and more balanced datasets could improve the generalizability of findings. Such research could enhance our understanding of brain lateralization, aiding in personalized approaches to education, rehabilitation, and treatment of language-related disorders.
